# Age modifies the association between severe sleep apnea and all-cause mortality

**DOI:** 10.1016/j.sleep.2024.06.012

**Published:** 2024-06-13

**Authors:** Amin Ramezani, Mehrnaz Azarian, Amir Sharafkhaneh, Arash Maghsoudi, Melissa B. Jones, Thomas Penzel, Javad Razjouyan

**Affiliations:** a Center for Innovations in Quality, Effectiveness, and Safety, Michael E. DeBakey VA Medical Center, Houston, TX, USA; b Department of Medicine, Baylor College of Medicine, Houston, TX, USA; c Pulmonary, Critical Care and Sleep Medicine Section, Medical Care Line, Michael E. DeBakey VA Medical Center, Houston, TX, USA; d Mental Health and Research Care Lines, Michael E. DeBakey VA Medical Center, Houston, TX, USA; e Menninger Department of Psychiatry and Behavioral Sciences, Baylor College of Medicine, Houston, TX, USA; f Sleep Medicine Center, Charite University Hospital Berlin, Berlin, Germany; g Big Data Scientist Training Enhancement Program (BD-STEP), VA Office of Research and Development, Washington, DC, USA

## Abstract

**Purpose:**

While sleep apnea (SA) gets more prevalent with advancing age, the impact of age on the association between SA and health outcomes is not well known. We assessed the association between the severity of SA and all-cause mortality in different age groups using large longitudinal data.

**Method:**

We applied a Natural Language Processing pipeline to extract the apnea-hypopnea index (AHI) from the physicians’ interpretation of sleep studies performed at the Veteran Health Administration (FY 1999–2022). We categorized the participants as no SA (n-SA, AHI< 5) and severe SA (s-SA, AHI≥30). We grouped the cohort based on age: Young≤40; Middle-aged:40–65; and Older adults≥65; and calculated the odds ratio (aOR) of mortality adjusted for age, sex, race, ethnicity, BMI, and Charlson-Comorbidity Index (CCI) using n-SA as the reference.

**Results:**

We identified 146,148 participants (age 52.23 ± 15.02; BMI 32.11 ± 6.05; male 86.7 %; White 66 %). Prevalence of s-SA increased with age. All-cause mortality was lower in s-SA compared to n-SA in the entire cohort (aOR,0.56; 95%CI: 0.54,0.58). Comparing s-SA to n-SA, the all-cause mortality rates (Young 1.86 % vs 1.49 %; Middle-aged 12.07 % vs 13.34 %; and Older adults 26.35 % vs 40.18 %) and the aOR diminished as the age increased (Young: 1.11, 95%CI: 0.93–1.32; Middle-aged: 0.64, 95%CI: 0.61–0.67; and Older adults: 0.44, 95%CI: 0.41–0.46).

**Conclusion:**

The prevalence of severe SA increased while the odds of all-cause mortality compared to n-SA diminished with age. SA may exert less harmful effects on the aged population. A causality analysis is warranted to assess the relationship between SA, aging, and all-cause mortality.

## 1. Introduction

The relation between the prevalence and severity of sleep apnea (SA) and all-cause mortality across different age groups is still debated. While some studies indicate a positive correlation between higher apnea-hypopnea index (AHI) and mortality [1–5], there is also evidence suggesting that certain degrees of intermittent hypoxia may confer a protective effect on survival [6,7]. Furthermore, the association between SA and adverse clinical outcomes (all-cause mortality, incident hypertension, cardiovascular and cerebrovascular diseases) may vary among different age groups [4], with higher rates observed in younger individuals [8–12]. Although most of these studies have shed light on this topic, none have exclusively examined the role of age in the relationship between severe SA and all-cause mortality compared to those with no SA, within a large cohort.

The severity of SA measured by AHI [13,14] is commonly documented as a note in the patient free-form text in the electronic medical records (EMR). Previously our team developed a natural language processing (NLP) pipeline to extract AHI from polysomnography notes using a rule-based algorithm [15]. In this paper on the technical front, we further developed the NLP pi hio9upeline by using a transformer-based language model to identify the AHI in the text and determine the correct value from any sleep study notes. We then used this extracted data to further investigate the relationship between severe SA and mortality. Unlike previous studies, the combination of the new NLP pipeline and the large, longitudinal national Veterans Health Administration (VHA) EMR allowed us to explore the complex relationship between severe SA and mortality while adjusting for potential confounders in large numbers of patient from a naturalistic setting [16–18]. We hypothesized that severe SA was associated with higher mortality compared to no SA, and that age modulates this mortality risk.

## 2. Methods

The project was approved by Baylor College of Medicine IRB (H-35366) and Michael E. DeBakey VA Medical Center (MEDVAMC) Research & Development committee.

### 2.1. Research design and participant data collection

This is a retrospective cohort study using electronic medical records (EMRs) from the VHA. The initial cohort included veterans referred to sleep disorders centers at the VHA from October 1, 1999, to September 30, 2022, with any sleep related ICD-9 or ICD-10 diagnostic codes and any sleep service Current Procedural Terminology (CPT) codes. For this study, we limited the cohort to individuals with medical visits associated with specific CPT codes, including ‘0203T,’ ‘0204T,’ ‘95782,’ ‘95783,’ ‘95800,’ ‘95801,’ ‘95806,’ ‘95808,’ ‘95810,’ ‘95811,’ ‘95819,’ ‘95821,’ ‘95822,’ ‘95827,’ ‘95828,’ ‘99508,’ ‘G0398,’ ‘G0399,’ and ‘G0400.’ Reports containing keywords in their titles, such as ‘%poly%,’ ‘%PSG%,’ ‘%sleep%,’ ‘%HST%,’ and ‘%HSAT%,’ were then selected. Subsequently, reports were further refined based on the presence of specific search terms, including ‘%apnea%,” ‘%hypopnea%’ and ‘%AHI%’. For patients with multiple visits, we considered the first visit documenting the AHI in the patient’s free-form text as the index date. We further limited the study population to individuals without positive airway pressure (PAP) treatment prior to the index date, as identified with CPT codes for positive airway pressure (PAP) treatment (‘94660′, ‘E0452’, ‘E0470’, ‘E0471’, ‘E0472’, ‘E0601’, ‘G8845’, ‘G8852’). The summary of the procedure is presented in strobe diagram (eFigure1).

### 2.2. Study variables

#### Outcome variables:

The primary outcome of interest was all-cause mortality. All-cause mortality data was gathered from the corporate data warehouse (CDW) VHA Vital Status table (sensitivity 98.3 % and specificity 99.8 % relative to the National Death Index) [19,20]. Mortality data was recorded up to August 23, 2023.

#### Exposure variable:

We used the level of AHI as the exposure variable. To have the largest effect size we focused on participants with severe SA (s-SA with AHI≥30) and no-SA (n-SA with AHI<5) [21]. We excluded those with AHI ≥5 and < 30 as the relationship between milder form of SA and all-cause mortality has not been shown in prior studies [5]. We excluded any participant with no SA with PAP prescription 1 year after the index date.

#### Other variables:

We collected patient demographics such as age (stratified to Young ≤40, Middle-age 40–65, and Older-adults ≥65 yrs), body mass index (BMI) (categorized to obese with BMI≥30), sex, race (white, black, and others), ethnicity (Hispanic). Using ICD9/10 codes for different comorbidities (Supplementary Table 4), we calculated Charlson Comorbidity Index (CCI), in addition to specific rates of each associated condition. The data on comorbid conditions were collected from inpatient or outpatient encounters within a year period before the index date [22]. Using PAP-related CPT codes, we calculated the proportion of patients with PAP prescriptions within one year after the index date. The follow-up time was extracted based on the duration between the index date of each participant until the time of death or the end of the study period (September 2022).

### 2.3. NLP method and validation for AHI

We developed a natural language processing (NLP) pipeline specifically designed to extract AHI data from unstructured notes obtained from physician interpretations of polysomnography (PSG) and home sleep testing (HST) done on the participants. After preprocessing the text, which included stemming, lemmatization, and tokenization, we utilized a pre-trained comprehensive language model (deepset-roberta-base-squad2 from Hugging Face hub) to generate concise summaries. The pipeline then identified segments containing relevant keywords, such as AHI, Apnea Hypopnea Index and Sleep Apnea. Subsequently, by formulating a query about the AHI quantity, the model extracted the corresponding values. To evaluate the effectiveness of the pipeline, we used a validation set consisting of 396 annotated notes created by experienced sleep physicians. These notes were categorized into two groups: s-SA (30 ≤ AHI ≤80) and n-SA (AHI <5). Our analysis revealed an overall accuracy rate of 87 % for the NLP algorithm’s ability to extract AHI information from these unstructured notes. Performance metrics and a heatmap were generated for these categories. The discriminant performance within each group surpassed 86 %, as illustrated in Tables S1 and S2, and between the two groups it exceeded 90 % as demonstrated in Table S3.

### 2.4. Statistical analysis

Using R software (RStudio Team (2020), RStudio: Integrated Development for R. RStudio, PBC, Boston, MA URL http://www.rstudio.com/), we implemented the Chi-square test for categorical variables and the student t-test for continuous variables. We reported the odds ratio (OR) and 95 % confidence intervals (95%CI) of patients with s-SA compared to the n-SA as reference. We adjusted the OR (aOR) for age, sex, race, ethnicity, BMI, and CCI. We performed a subgroup analysis based on the age groups. We calculated mortality rates for the entire cohort with a weighted average considering the size of each age group. A p value < 0.05 was considered statistically significant. We used Kaplan–Meier curves to demonstrate the relationship between SA categories and survival rate among three age groups. We utilized proportional hazards regression models to compute unadjusted and adjusted relative hazard ratios (HRs) for all-cause mortality. Covariates considered individually and collectively included age, sex, race, ethnicity, BMI, and CCI.

## 3. Results

The analysis encompassed a cohort of 146,148 participants (average age, 52.23 ± 15.02; average BMI, 32.11 ± 6.05; 86.7 % male; 66 % white; average CCI, 1.02 ± 1.69; and proportion with CCI ≥2, 23.1 %) (Table 1). The s-SA category was significantly older (56.7 ± 13.7) compared to n-SA (45.7 ± 14.5). This group also had significantly higher BMI (33.47 ± 6.1) and CCI (1.26 ± 1.86) compared to n-SA (BMI, 30.14 ± 5.41; CCI, 0.68 ± 1.34). The overall weighted average rates of mortality in the entire cohort, n-SA and s-SA were 13.61 %, 11.55 %, and 15.03 % respectively. The mean follow-up times of the entire cohort, n-SA and s-SA were 5.49 (±4.56), 6.35 (±4.63), and 4.9 (±4.42) respectively.

Table 2 provides the baseline characteristics and all-cause mortality rates stratified by age and AHI level. Among all age groups, the rate of BMI≥30 was higher in s-SA category compared to n-SA (72.5 % vs. 46 % in Young; 74.2 % vs. 48.1 % in Middle-aged group and 61.4 % vs. 41.5 % in Older-adults). Moreover, the proportion of participants with CCI≥2 in s-SA compared to n-SA category was higher among Young (3.8 % vs. 2.7 %), Middle-aged (23.5 % vs. 18.3 %) and Older-adults (49.7 % vs. 46.1 %). The rate of insomnia was higher among those in n-SA category compared to s-SA category in all age groups (27.1 % vs. 21.7 % in Young, 22.2 % vs. 15.7 % in Middle-aged group, and 17.5 % vs. 14.2 % in Older-adults). The rates of cardiovascular disorders in s-SA compared to n-SA category were higher in Young (21.3 % vs 15.23 %) and Middle-aged (61.06 % vs 57.33 %) but similar among Older-adults (86.1 % vs 85.1 %). Those with severe SA, compared to those with no SA, had lower rates of pulmonary disorders in Middle-aged (23.35 % vs 27.53 %) and Older adults (35.07 % vs 46.27 %) while the rates were similar in Young (11.61 % vs11.69 %). Metabolic and renal disorders were more prevalent in s-SA category compared to n-SA category among all ages. The proportion of those with s-SA who received PAP treatment increased with advancing age (53.16 % in Young, 60.58 % in Middle-aged, and 62.55 % in Older-adults).

**Fig. 1.**
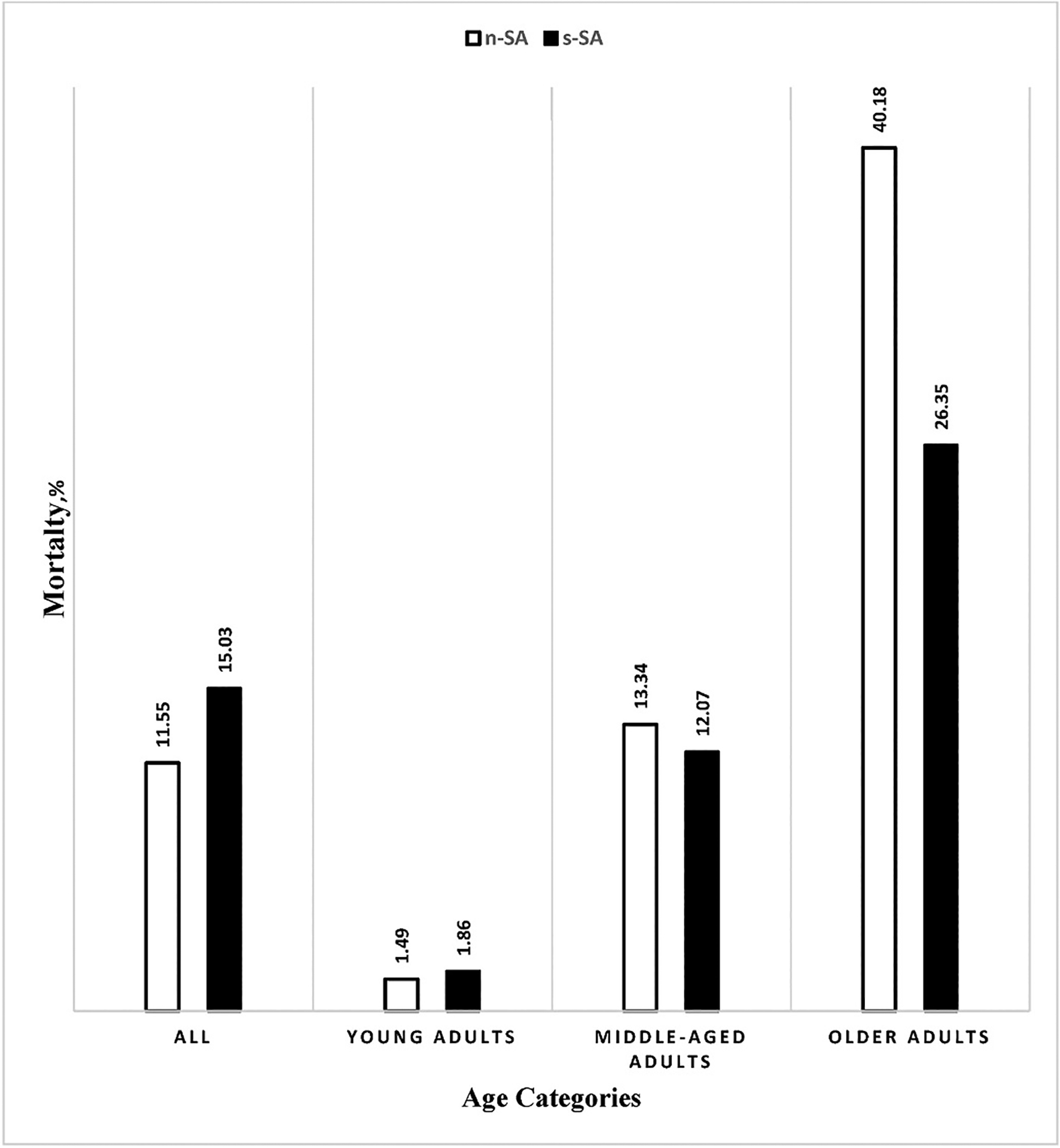
All-cause mortality rates for different age and AHI categories “Comparing each variable between n-SA and s-SA category demonstrated significant p-values (P-value ≤0.05) among all items.”

Fig. 1 represents the all-cause mortality rates for the entire cohort and for the age groups. The all-cause mortality rates in both s-SA and n-SA increased with advancing age. However, all-cause mortality rates differed across the age groups in s-SA versus n-SA. Among the Young adults, all-cause mortality was higher in s-SA category compared to n-SA (1.86 % vs. 1.49 %, p < 0.001). The middle-aged group exhibited slightly lower all-cause mortality for s-SA compared to n-SA (12.07 % vs. 13.34 %, p < 0.001). In the Older adult group, the mortality rate for s-SA compared to n-SA was markedly lower (26.35 % vs. 40.18 %, p < 0.001).

Raw and adjusted odds ratios (the ratio of odds of an event in one group versus the odds of the event in the other group) for all-cause mortality are presented in Table 3. Initially, s-SA was associated with increased mortality in the entire cohort (Unadjusted OR: 1.35, 95 % CI:1.31,1.4). After adjusting for age, gender, ethnicity, BMI, and CCI, this relationship was reversed, and s-SA was associated with a 44 % lower risk of all-cause mortality (aOR: 0.56, 95 % CI:0.54,0.58). When we further analyzed according to age groups, s-SA was associated with increased mortality in Young adults (Unadjusted OR: 1.25, 95 % CI: 1.06,1.47). This increase was no longer significant after adjusting for demographics and comorbidities (aOR: 1.11, 95 % CI: 0.933–1.32). On the other hand, among Middle-aged group and Older adults, s-SA was significantly associated with lower all-cause mortality and the association remained significant after adjusting for confounding variables (Middle-aged: aOR: 0.64, 95 % CI: 0.61, 0.67; Older adults: aOR: 0.44, 95 % CI: 0.41, 0.46). Additionally, we calculated the adjusted OR separately in subgroups of participants whose diagnoses were based on PSG versus HST for the entire cohort and each age group. The results are reported in Supplementary Table 5 and showed that the aORs regardless of diagnoses with either PSG or HST remained generally in the same direction as that of the entire cohort.

The raw and adjusted hazard ratios (the ratio of risk of an event in one group, versus the risk of the event in the other group) for the entire cohort and different age groups are summarized in Table 4. After adjusting for confounding factors, the HR among all ages showed better survival of s-SA compared to n-SA (aHR, 0.62; 95 % CI: 0.60, 0.64).

**Fig. 2.**
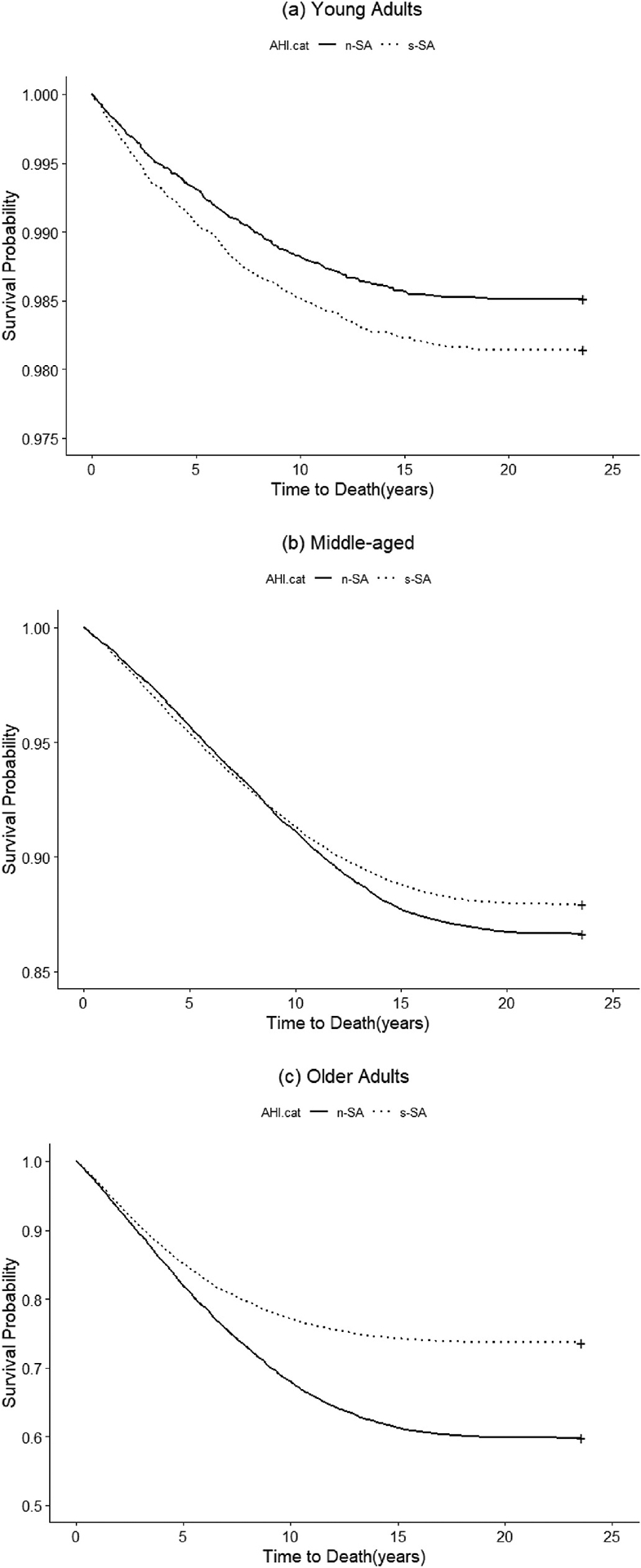
Kaplan-Meier survival curves across different categories of AHI for different age groups.

Fig. 2 presents Kaplan-Meier survival curves, illustrating the differences between the two SA categories for different age groups. In the Young adults (Fig. 2a), survival was lower in s-SA compared to n-SA (Unadjusted HR,1.25; 95%CI:1.06–1.47), while in the Middle-aged adults (Fig. 2b), survival was slightly higher in s-SA compared to n-SA (Unadjusted HR,0.90; 95%CI:0.87–0.94). Moreover, in the Older-adults (Fig. 2c), s-SA compared to n-SA, was significantly associated with higher survival (Unadjusted HR,0.62; 95%CI: 0.59–0.65). However, after adjusting for several forementioned confounding factors, the pattern for all-cause mortality among Young adults was not anymore significant (aHR, 1.11 95 % CI: 0.93, 1.32), while it remained significant for the Middle-aged (aHR, 0.64; 95 % CI: 0.61, 0.67) and the Older adults (aHR, 0.44 95 %; CI: 0.41,0.46)

## 4. Discussion

We conducted a retrospective study on a large cohort of Veterans with available AHI data extracted using an innovative and unique NLP pipeline. While the prevalence of severe SA increased with advancing age, the rate, and odds of all-cause mortality in those with severe sleep apnea declined in comparison to participants with no SA. The reduced odds of all-cause mortality among middle-aged and older adults remained significant after adjusting for possible confounding factors.

The role of age in determining the relationship between sleep apnea and all-cause mortality, while having great clinical and public health significance, is still debated. Consistent with the literature, the rate of all-cause mortality and the prevalence of severe SA increased with age in our study cohort [3,8,9,23–26]. Our data showed an interesting reversal in all-cause mortality difference between severe SA and no SA with advancing age. This difference was specifically more pronounced among the older adults (age≥65). It is noteworthy that our results are similar to findings related to other chronic medical conditions with all-cause mortality: as the number of chronic medical conditions accumulate with age [27], the detrimental effect of any individual condition on survival may decline [28]. The modifying role of age in the association between SA and mortality was also investigated in previous studies and has shown conflicting results. In some way, data from Sleep Heart Health Study was in line with our study, showing that SA was not associated with increased mortality in subjects over 70 years of age [3, 29]. Their results showed that the adjusted hazard ratios for all-cause mortality associated with severe SA (AHI≥30) compared to no SA was not significant. This finding, while different from our findings, does not contradict ours. Our data suggests that older adults with severe SA may be protected against adverse outcomes.

The pathophysiological protective effect of SA in older adults might be direct or indirect. Sleep apnea is associated with intermittent hypoxia (IH), and recent data suggest that IH may provide protective advantages during and after acute vital organ injuries such as acute MI and stroke [7,30], which according to CDC are two of the major causes of death in older people [31]. This phenomenon, known as ischemic or hypoxic preconditioning (HP), occurs when chronic IH is administered below the threshold of damage, resulting in increased tolerance to severe acute ischemic exposure [32]. Alternatively, treatment of SA may positively impact clinical outcomes in old adults compared to young group and presence of SA exerts an indirect protective effect on mortality. Further, some studies showed motivation to using and adhering to PAP increases with advancing age [33,34]. Our data clearly showed an increase in proportion of PAP prescription with age.

On the other hand, our results indicated a higher mortality rate among veterans with severe SA compared to those with no SA in people younger than 40. In this regard, Lavie et al. and Marti et al. have also demonstrated that severe SA is associated with excess mortality in the general population, but only in the group of younger than 50 [10,23]. Moreover, the more prominent association between SA severity and all-cause mortality in people younger than 50 was shown in two other studies by He et al. and Rich et al. [8,9]. However, adjustment for demographics and medical comorbidities reduced the strength of the relationship between severe SA and excess mortality. This suggests that other comorbid conditions may be confounding the increased risk of mortality associated with severe SA in this age group. The heightened mortality seen in younger individuals could be linked to various factors. Generally, younger population have fewer comorbid conditions. Further, the primary cause of death among those under 44 is accidental injuries [35] which can be increased due to excessive daytime sleepiness related induced by SA. For instance, the well-established association between SA and an increased likelihood of motor vehicle accidents in the general population supports this notion [36,37]. Moreover, it is known that the incentive to regular usage of PAP is lower among younger adults [33,34].Effect of SA on mortality is more prominent in younger age due to fewer comorbid conditions competing to cause death in younger group. In contrast, among old adult group, other comorbidities causing death start to be more abundant and have higher effect on mortality, so that SA plays a less prominent role. However, we found that after adjusting for CCI, older people with severe SA still had lower risk of all-cause mortality compared to those without SA.

Our data show an overall higher rate of all-cause mortality in patients with severe SA compared to those without SA. After adjusting for the confounding factors including age, the relationship reversed. The differential effect of SA on risk of incident CVD or HTN only among young and middle-aged adults maybe one of the likely explanations [11, 12]. The Busselton Health Study [38] and Wisconsin Sleep Cohort [5] reported adjusted hazard ratios of 6.24 and 2.7 for patients with severe SA compared to no SA, respectively. However, the mean age and age range of their population was lower than our overall cohort. The age values in Busselton and Wisconsin cohort were 53.06 (range of 40–65) and 49.0 (range of 30–60), respectively. Therefore, the findings in the two studies are consistent with the younger group in our study. The differential effect of SA on all-cause mortality in different age groups may stem from their vulnerability to survivor bias. Finally, it is tempting to speculate that the age decline in relative mortality results from the fact that most people with sleep apnea successfully adapt to the nightly apneic events by an as yet unknown mechanism [10] or adapt to nightly use of CPAP(33, 34).

The findings of our study are novel and have several strengths. We leveraged a well-validated, AI-based NLP to extract data from the medical record in a large and naturalistic cohort of Veterans (almost 150,000). The number of subjects and duration of follow up dominated many other previous cohorts. The NLP pipeline also enabled us to categorize SA severity according to the AHI. In contrast to community-based, prospective cohort studies, we focused specifically on participants drawn from sleep centers within VHA. Our cohort was diverse in terms of age, BMI, CCI, ethnicity, and race. This study utilized data from veterans enrolled in the VHA from 2000 to 2022 and VA healthcare centers from across the country.

Our study has limitations. The first limitation is the restricted variants in sleep report and examination: We only used AHI parameter to diagnose SA and we did not have data on hypoxia and its severity on this cohort. In addition, we did not have access to raw data and how the hypopneas were defined in each sleep study. Our no sleep apnea category may not be a true control as they were participants referred to VHA sleep centers for sleep related problems. Also, almost 87 % of population are male, which induces gender-biases in study. This bias reflects the gender distribution among Veterans receiving healthcare at the VA and should be considered when interpreting the study’s findings. Another limitation is the dependance on annotated documents which may be impacted by human error. Incorrectness influences the training rules and learning process of NLP tasks. Also, the low number of annotated notes further degrades the NLP output reliability. Moreover, the lack of data regarding the cause of death in our cohort should not be over-looked. A dedicated exploration of specific considerations and implications for female participants and addition of cause of death is warranted in subsequent studies to address this identified gap in the existing literature. Further studies for finding a cut-off point and range of AHI in which people with severe SA experience lower rate of mortality is also necessary and can have several clinical implications. To better understand the role of SA treatment on the association between SA severity and all-cause mortality, finding the dosage of PAP treatment for each participant and adjusting the results based on PAP usage would be beneficial.

## 5. Conclusion

The relationship between severe SA and all-cause mortality is influenced by age. While severe sleep apnea was associated with increased rate and odds of all-cause mortality among younger population, this association reversed with increasing age. Among Middle-aged and Older adults, severe sleep apnea shows protective behavior against all-cause mortality. Finding the exact pathophysiological mechanism underlying this phenomenon should be further investigated.

## Supplementary Material

Supplementary

## Figures and Tables

**Table 1:** Baseline characteristics of the Sleep Study cohort by Apnea-Hypopnea Index (AHI) category.

	All Participants	n-SA (AHI <5)	s-SA (AHI ≥30)
**N (%)**	146,148	59,707	86,441
**Sex (Male) N (%)**	126,814 (86.77)	46,768 (78.33)	80,046 (92.6)
**Ethnicity (Hispanic)N (%)**	13,774 (9.42)	5088 (8.52)	8686 (10.05)
**Race**			
White, N (%)	96,476 (66.01)	37,919 (63.51)	58,557 (67.74)
Black, N (%)	34,632 (23.7)	16,018 (26.83)	18,614 (21.53)
Others, N (%)	15,040 (10.29)	5770 (9.66)	9270 (10.72)
**Age, M(SD)**	52.23 (15.02)	45.77 (14.5)	56.69 (13.69)
Young (Age≤40) N (%)	38,723 (26.5)	25,522 (42.75)	13,201 (15.27)
Middle age (40<Age<65) N (%)	72,769 (49.79)	26,901 (45.06)	45,868 (53.06)
Elderly (Age≥65) N (%)	34,656 (23.71)	7284 (12.2)	27,372 (31.67)
**BMI, M(SD)**	32.11 (6.05)	30.14 (5.41)	33.47 (6.1)
**BMI≥ 30, N (%)**	88,159 (60.32)	27,721 (46.43)	60,438 (69.92)
**CCI, M(SD)**	1.02 (1.69)	0.68 (1.34)	1.26 (1.86)
**CCI≥ 2, N (%)**	33,869 (23.17)	8981 (15.04)	24,888 (28.79)
**Death, N (%)**	19,890 (13.61)	6896 (11.55)	12,994 (15.03)
**Follow-up time, M (SD)**	5.49 (4.56)	6.35 (4.63)	4.9 (4.42)

“Comparing each variable between n-SA and s-SA category demonstrated significant p-values (P-value ≤0.05) among all items.”

**Table 2:** Mortality rates and clinical characteristics stratified by age and AHI categories.

Age group	Young Adults		Middle-aged		Older Adults	
AHI Category	n-SA	s-SA	n-SA	s-SA	n-SA	s-SA
N	25,522	13,201	26,901	45,868	7284	27,372
AHI, M(SD)	1.97 (1.34)	45.68 (16.32)	2.13 (1.38)	51.29 (16.6)	2.22 (1.39)	52.89 (17.54)
BMI (≥30), N (%)	11,744 (46.02)	9575 (72.53)	12,955 (48.16)	34,060 (74.26)	3022 (41.49)	16,803 (61.39)
CCI (≥2), N (%)	692 (2.71)	498 (3.77)	4927 (18.32)	10,772 (23.48)	3362 (46.16)	13,618 (49.75)
Insomnia^¤^, N (%)	6925 (27.13)	2862 (21.68)	6162 (22.91)	7234 (15.77)	1275 (17.5)	3884 (14.19)
Cardiovascular^¤^, N (%)	4143 (16.23)	2812 (21.3)	15,423 (57.33)	28,005 (61.06)	6276 (86.16)	23,301 (85.13)
Metabolic^¤^, N (%)	5788 (22.68)	4845 (36.7)	16,577 (61.62)	33,354 (72.72)	6131 (84.17)	24,061 (87.9)
Neurologic^¤^, N (%)	4302 (16.86)	1880 (14.24)	6282 (23.35)	8065 (17.58)	2171 (29.81)	7153 (26.13)
Psychiatry^¤^, N (%)	19,087 (74.79)	9608 (72.78)	17,499 (65.05)	26,895 (58.64)	3403 (46.72)	12,956 (47.33)
Renal^¤^, N (%)	1019 (3.99)	762 (5.77)	3728 (13.86)	8226 (17.93)	2221 (30.49)	9840 (35.95)
Pulmonary^¤^, N (%)	2963 (11.61)	1543 (11.69)	7407 (27.53)	10,708 (23.35)	3370 (46.27)	9599 (35.07)
PAP, N (%)	0	7018 (53.16)	0	27,786 (60.58)	0	17,121 (62.55)
Death, N (%)	380 (1.49)	245 (1.86)	3589 (13.34)	5537 (12.07)	2927 (40.18)	7212 (26.35)

“Comparing each variable between n-SA and s-SA category demonstrated significant p-values (P-value ≤0.05) among all items.”

ICD9/10 codes were used for extracting comorbidities from inpatient and outpatient notes within one year prior to index date.

**Table 3:** Adjusted Odds Ratios (95 % confidence intervals) for all-cause mortality associated with sleep apnea.

All ages	Unadjusted OR (95%CI)	Adj. OR (95%CI)
n-SA (AHI <5)	REF	REF
s-SA (AHI ≥30)	1.35 (1.31,1.4)	0.56 (0.54, 0.58)
**Age ≤ 40**		
n-SA (AHI <5)	REF	REF
s-SA (AHI ≥30)	1.25 (1.06,1.47)	1.11 (0.93, 1.32)
**40<age<65**		
n-SA (AHI <5)	REF	REF
s-SA (AHI ≥30)	0.89 (0.85,0.93)	0.64 (0.61, 0.67)
**Age ≥ 65**		
n-SA (AHI <5)	REF	REF
s-SA (AHI ≥30)	0.53 (0.5,0.56)	0.44 (0.41, 0.46)

Adjusted for age, sex, race, ethnicity, BMI and CCI.

**Table 4:** Adjusted Hazard Ratios (95 % confidence intervals) for all-cause mortality associated with sleep apnea in the VA Health Study.

All ages	Unadjusted HR (95%CI)	Adj. HR (95%CI)
n-SA (AHI <5)	REF	REF
s-SA (AHI ≥30)	1.34 (1.30,1.38)	0.62 (0.60,0.64)
**Age ≤ 40**		
n-SA (AHI <5)	REF	REF
s-SA (AHI ≥30)	1.25 (1.06,1.47)	1.11 (0.93,1.32)
**40<age<65**		
n-SA (AHI <5)	REF	REF
s-SA (AHI ≥30)	0.90 (0.87,0.94)	0.68 (0.66,0.72)
**Age ≥ 65**		
n-SA (AHI <5)	REF	REF
s-SA (AHI ≥30)	0.62 (0.59,0.65)	0.54 (0.52,57)

Adjusted for age, sex, race, ethnicity, BMI and CCI.
