# Supplementary material for: Age modifies the association between severe sleep apnea and all-cause mortality
Source: Sleep Med. Author manuscript; Available in PMC 2025 Sep 1. (PMC11385665; doi:10.1016/j.sleep.2024.06.012)
Supplement: Supplementary [file NIHMS2021292-supplement-Supplementary.docx]

# Supplements:

**eFigure1. STROBE diagram for the population selection in this study.**

Veterans with any sleep diagnosis or services from 10/1999 to 09/2022,

N = 4,837,321

Veterans without related CPT codes,

N = 3,322,296

Veterans with related CPT codes,

N = 1,515,025

Veterans without sleep related notes,

N = 244,360

Veterans with sleep related notes,

N = 1,270,665

Veterans without full information on AHI keywords,

N= 841,660

Veterans with full information on AHI keywords,

N = 429,005

Veterans with CPAP treatment before the index date,

N=33,705

Veterans without CPAP at the index date,

N = 395,300

Veterans with 5≤AHI<30,

N=199,992

Veterans with AHI<5 or AHI≥30,

N = 195,308

Veterans with no SA who used CPAP treatment 1 year after index date,

N=49,160

Final cohort

(All Veterans with severe SA and those with no SA and no CPAP treatment 1 year after index date),

N = 146,148

**Table S1: Performance Metrices in Discriminating different levels of test results. (Confusion Matrix)**

| AHI_Range | | NLP Algorithm value | | |
| --- | --- | --- | --- | --- |
|  |  | **s-SA**  **(30≤AHI ≤80)** | **n-SA**  **(AHI ≤5)** | **Total** |
| Actual Value  (Gold-Standard) | **s-SA**  **(30≤ AHI ≤80)** | **136** | **11** | **147** |
|  | **n-SA**  **(AHI ≤5)** | **7** | **242** | **249** |
|  | **Total** | **143** | **253** | **396** |

**Table S2: Performance Metrices in Discriminating different levels of test results. (Heatmap) (1: AHI≤5, 2: 30≤AHI≤80,)**


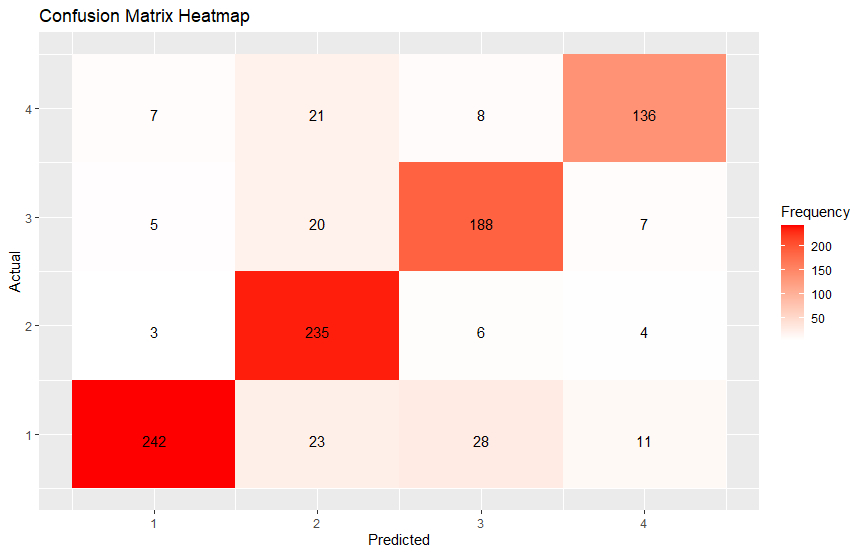


**Table S3: Performance Metrices (Positives vs Negatives)**

|  | AHI ≤ 5 | 30≤ AHI ≤80 |
| --- | --- | --- |
| Recall (Sensitivity) | **92%** | **89%** |
| Specificity (selectivity) | **88%** | **91%** |
| PPV (Precision) | **89%** | **86%** |
| NPV | **90%** | **90%** |
| F1 | **90%** | **88%** |
| Accuracy | **87%** | **86%** |

| **Table S4. List of ICD-9 and ICD-10 codes used for extracting different comorbidities.** | | |
| --- | --- | --- |
| **Comorbidities** | **ICD-9 codes** | **ICD-10 codes** |
| ***Insomnia*** | '307.42', '327.00', '327.01', '327.02', '327.09', '780.51', '780.52' | 'F51.01', 'F51.03', 'F51.04', 'F51.05', 'F51.09', 'G47.00', 'G47.01', 'G47.09' |
| **Cardiovascular Disorders** | | |
| ***Acute Cardiac Injury*** | '410’,'410’,'410.01’,'410.02’,'410.1’,'410.1’,'410.11’,'410.12’,'410.2’,'410.2’,'410.21’,'410.22’,'410.3’,'410.3’,'410.31’,'410.32’,'410.4’,'410.4’,'410.41’,'410.42’,'410.5’,'410.5’,'410.51’,'410.52’,'410.6’,'410.6’,'410.61’,'410.62’,'410.7’,'410.7’,'410.71’,'410.72’,'410.8’,'410.8’,'410.81’,'410.82’,'410.9’,'410.9’,'410.91’,'410.92’,'861’,'861.01’,'861.02’,'861.03’,'861.1’,'861.11’,'861.12’,'861.13’, | 'I21.01’,'I21.02’,'I21.09’,'I21.11’,'I21.19’,'I21.21’,'I21.29’,'I21.3’,'I21.4’,'I21.9’,'I21.A1’,'I21.A9’,'S26.00XA’,'S26.00XD’,'S26.00XS’,'S26.01XA’,'S26.01XD’,'S26.01XS’,'S26.020A’,'S26.020D’,'S26.020S’,'S26.021A’,'S26.021D’,'S26.021S’,'S26.022A’,'S26.022D’,'S26.022S’,'S26.09XA’,'S26.09XD’,'S26.09XS’,'S26.10XA’,'S26.10XD’,'S26.10XS’,'S26.11XA’,'S26.11XD’,'S26.11XS’,'S26.12XA’,'S26.12XD’,'S26.12XS’,'S26.19XA’,'S26.19XD’,'S26.19XS’,'S26.90XA’,'S26.90XD’,'S26.90XS’,'S26.91XA’,'S26.91XD’,'S26.91XS’,'S26.92XA’,'S26.92XD’,'S26.92XS’,'S26.99XA’,'S26.99XD’,'S26.99XS’, |
| ***Acute Myocardial Infarction*** | '410’,'410’,'410.01’,'410.02’,'410.1’,'410.1’,'410.11’,'410.12’,'410.2’,'410.2’,'410.21’,'410.22’,'410.3’,'410.3’,'410.31’,'410.32’,'410.4’,'410.4’,'410.41’,'410.42’,'410.5’,'410.5’,'410.51’,'410.52’,'410.6’,'410.6’,'410.61’,'410.62’,'410.7’,'410.7’,'410.71’,'410.72’,'410.8’,'410.8’,'410.81’,'410.82’,'410.9’,'410.9’,'410.91’,'410.92’, | 'I21.01’,'I21.02’,'I21.09’,'I21.11’,'I21.19’,'I21.21’,'I21.29’,'I21.3’,'I21.4’,'I21.9’,'I21.A1’,'I21.A9’, |
| ***Arrhythmia*** | '427.6’,'427.69’,'427.89’,'427.9’, | 'I47.0’,’I49.8’,’I49.9’, |
| ***Coronary Atherosclerotic Heart Disease*** | '391.1’,'391.2’,'391.8’,'391.9’,'411.8’,'411.89’,'413.9’,'414.01’,'414.06’,'414.3’,'414.4’,'414.8’,'414.9’,'425.1’,'425.11’,'425.18’,'425.2’,'425.7’,'425.8’,'425.9’,'440.1’,'440.2’,'440.21’,'440.22’,'440.23’,'440.24’,'440.29’,'440.8’,'440.9’,'V17.3’,'V81.0’, | 'I20.0’,’I20.1’,’I20.8’,'I20.9’,’I24.0’,’I24.8’,’I24.9’,’I25.10’,’I25.110’,'I25.111’,'I25.18’,’I25.119’,'I25.2’,'I25.5’,'I25.6’,'I25.700’,'I25.701’,'I25.708’,'I25.709’,'I25.710’,'I25.711’,'I25.718’,'I25.719’,'I25.720’,'I25.721’,'I25.728’,'I25.729’,'I25.730’,'I25.731’,'I25.738’,'I25.739’,'I25.750’,'I25.751’,'I25.758’,'I25.759’,'I25.760’,'I25.761’,'I25.768’,'I25.769’,'I25.790’,'I25.791’,'I25.798’,'I25.799’,'I25.810’,'I25.811’,'I25.812’,'I25.82’,'I25.83’,'I25.84’,'I25.89’,'I25.9’,'Z95.1’,'Z95.5’,'Z98.61’, |
| ***Cardiomyopathy*** | '74.21’,'93.81’,'98.83’,'391’,'420’,'420.9’,'420.91’,'420.99’,'423.1’,'423.2’,'425’,'425.1’,'425.11’,'425.18’,'425.2’,'425.3’,'425.4’,'425.5’,'425.7’,'425.8’,'425.9’, | 'A36.81’,'A38.1’,'A39.50’,'A39.52’,'B26.82’,'B33.20’,'B33.22’,'B33.23’,'B33.24’,'B58.81’,'I41.’,'I42.0’,'I42.1’,'I42.2’,'I42.3’,'I42.4’,'I42.5’,'I42.6’,'I42.8’,'I42.9’,'I43.’, |
| ***Congestive Heart Failure*** | '428’, | 'I50.20’,'I50.21’,'I50.22’,'I50.23’,'I50.30’,'I50.31’,'I50.32’,'I50.33’,'I50.40’,'I50.41’, |
| ***Cardiovascular disease*** | '410’,'410’,'410.01’,'410.02’,'410.1’,'410.1’,'410.11’,'410.12’,'410.2’,'410.2’,'410.21’,'410.22’,'410.3’,'410.3’,'410.31’,'410.32’,'410.4’,'410.4’,'410.41’,'410.42’,'410.5’,'410.5’,'410.51’,'410.52’,'410.6’,'410.6’,'410.61’,'410.62’,'410.7’,'410.7’,'410.71’,'410.72’,'410.8’,'410.8’,'410.81’,'410.82’,'410.9’,'410.9’,'410.91’,'410.92’,'411’,'411.1’,'411.8’,'411.81’,'411.89’,'412’,'414’,'414’,'414.01’,'414.02’,'414.03’,'414.04’,'414.05’,'414.06’,'414.07’,'414.2’,'414.3’,'414.4’,'414.8’,'414.9’,'428’,'428.1’,'428.2’,'428.21’,'428.22’,'428.23’,'428.3’,'428.31’,'428.32’,'428.33’,'428.4’,'428.41’,'428.42’,'428.43’,'428.9’,'433’,'433’,'433.01’,'433.1’,'433.1’,'433.11’,'433.2’,'433.2’,'433.21’,'433.3’,'433.3’,'433.31’,'433.8’,'433.8’,'433.81’,'433.9’,'433.9’,'433.91’,'434.1’,'434.11’,'434.91’,'435’,'435.1’,'435.2’,'435.3’,'435.8’,'435.9’,'440’,'440.1’,'440.2’,'440.2’,'440.21’,'440.22’,'440.23’,'440.24’,'440.29’,'440.3’,'440.31’,'440.32’,'440.4’,'440.8’,'440.9’,'441’,'441’,'441.01’,'441.02’,'441.03’,'441.1’,'441.2’,'441.3’,'441.4’,'441.5’,'441.6’,'441.7’,'441.9’,'V45.81’,'V45.82’, | 'G45.9’,'I20.0’,'I20.1’,'I20.8’,'I20.9’,'I21.01’,'I21.02’,'I21.09’,'I21.11’,'I21.19’,'I21.21’,'I21.29’,'I21.3’,'I21.4’,'I21.9’,'I21.A1’,'I21.A9’,'I22.0’,'I22.1’,'I22.2’,'I22.8’,'I22.9’,'I24.0’,'I24.1’,'I24.8’,'I24.9’,'I25.10’,'I25.110’,'I25.111’,'I25.118’,'I25.119’,'I25.2’,'I25.3’,'I25.41’,'I25.42’,'I25.5’,'I25.6’,'I25.700’,'I25.701’,'I25.708’,'I25.709’,'I25.710’,'I25.711’,'I25.718’,'I25.719’,'I25.720’,'I25.721’,'I25.728’,'I25.729’,'I25.730’,'I25.731’,'I25.738’,'I25.739’,'I25.750’,'I25.751’,'I25.758’,'I25.759’,'I25.760’,'I25.761’,'I25.768’,'I25.769’,'I25.790’,'I25.791’,'I25.798’,'I25.799’,'I25.810’,'I25.811’,'I25.812’,'I25.82’,'I25.83’,'I25.84’,'I25.89’,'I25.9’,'I50.1’,'I50.20’,'I50.21’,'I50.22’,'I50.23’,'I50.30’,'I50.31’,'I50.32’,'I50.33’,'I50.40’,'I50.41’,'I50.42’,'I50.43’,'I50.810’,'I50.811’,'I50.812’,'I50.813’,'I50.814’,'I50.82’,'I50.83’,'I50.84’,'I50.89’,'I50.9’,'I63.00’,'I63.011’,'I63.012’,'I63.013’,'I63.019’,'I63.02’,'I63.031’,'I63.032’,'I63.033’,'I63.039’,'I63.09’,'I63.10’,'I63.111’,'I63.112’,'I63.113’,'I63.119’,'I63.12’,'I63.131’,'I63.132’,'I63.133’,'I63.139’,'I63.19’,'I63.20’,'I63.211’,'I63.212’,'I63.213’,'I63.219’,'I63.22’,'I63.231’,'I63.232’,'I63.233’,'I63.239’,'I63.29’,'I63.30’,'I63.311’,'I63.312’,'I63.313’,'I63.319’,'I63.321’,'I63.322’,'I63.323’,'I63.329’,'I63.331’,'I63.332’,'I63.333’,'I63.339’,'I63.341’,'I63.342’,'I63.343’,'I63.349’,'I63.39’,'I63.40’,'I63.411’,'I63.412’,'I63.413’,'I63.419’,'I63.421’,'I63.422’,'I63.423’,'I63.429’,'I63.431’,'I63.432’,'I63.433’,'I63.439’,'I63.441’,'I63.442’,'I63.443’,'I63.449’,'I63.49’,'I63.50’,'I63.511’,'I63.512’,'I63.513’,'I63.519’,'I63.521’,'I63.522’,'I63.523’,'I63.529’,'I63.531’,'I63.532’,'I63.533’,'I63.539’,'I63.541’,'I63.542’,'I63.543’,'I63.549’,'I63.59’,'I63.6’,'I63.8’,'I63.81’,'I63.89’,'I63.9’,'I65.01’,'I65.02’,'I65.03’,'I65.09’,'I65.1’,'I65.21’,'I65.22’,'I65.23’,'I65.29’,'I65.8’,'I65.9’,'I70.0’,'I70.1’,'I70.201’,'I70.202’,'I70.203’,'I70.208’,'I70.209’,'I70.211’,'I70.212’,'I70.213’,'I70.218’,'I70.219’,'I70.221’,'I70.222’,'I70.223’,'I70.228’,'I70.229’,'I70.231’,'I70.232’,'I70.233’,'I70.234’,'I70.235’,'I70.238’,'I70.239’,'I70.241’,'I70.242’,'I70.243’,'I70.244’,'I70.245’,'I70.248’,'I70.249’,'I70.25’,'I70.261’,'I70.262’,'I70.263’,'I70.268’,'I70.269’,'I70.291’,'I70.292’,'I70.293’,'I70.298’,'I70.299’,'I70.301’,'I70.302’,'I70.303’,'I70.308’,'I70.309’,'I70.311’,'I70.312’,'I70.313’,'I70.318’,'I70.319’,'I70.321’,'I70.322’,'I70.323’,'I70.328’,'I70.329’,'I70.331’,'I70.332’,'I70.333’,'I70.334’,'I70.335’,'I70.338’,'I70.339’,'I70.341’,'I70.342’,'I70.343’,'I70.344’,'I70.345’,'I70.348’,'I70.349’,'I70.35’,'I70.361’,'I70.362’,'I70.363’,'I70.368’,'I70.369’,'I70.391’,'I70.392’,'I70.393’,'I70.398’,'I70.399’,'I70.401’,'I70.402’,'I70.403’,'I70.408’,'I70.409’,'I70.411’,'I70.412’,'I70.413’,'I70.418’,'I70.419’,'I70.421’,'I70.422’,'I70.423’,'I70.428’,'I70.429’,'I70.431’,'I70.432’,'I70.433’,'I70.434’,'I70.435’,'I70.438’,'I70.439’,'I70.441’,'I70.442’,'I70.443’,'I70.444’,'I70.445’,'I70.448’,'I70.449’,'I70.45’,'I70.461’,'I70.462’,'I70.463’,'I70.468’,'I70.469’,'I70.491’,'I70.492’,'I70.493’,'I70.498’,'I70.499’,'I70.501’,'I70.502’,'I70.503’,'I70.508’,'I70.509’,'I70.511’,'I70.512’,'I70.513’,'I70.518’,'I70.519’,'I70.521’,'I70.522’,'I70.523’,'I70.528’,'I70.529’,'I70.531’,'I70.532’,'I70.533’,'I70.534’,'I70.535’,'I70.538’,'I70.539’,'I70.541’,'I70.542’,'I70.543’,'I70.544’,'I70.545’,'I70.548’,'I70.549’,'I70.55’,'I70.561’,'I70.562’,'I70.563’,'I70.568’,'I70.569’,'I70.591’,'I70.592’,'I70.593’,'I70.598’,'I70.599’,'I70.601’,'I70.602’,'I70.603’,'I70.608’,'I70.609’,'I70.611’,'I70.612’,'I70.613’,'I70.618’,'I70.619’,'I70.621’,'I70.622’,'I70.623’,'I70.628’,'I70.629’,'I70.631’,'I70.632’,'I70.633’,'I70.634’,'I70.635’,'I70.638’,'I70.639’,'I70.641’,'I70.642’,'I70.643’,'I70.644’,'I70.645’,'I70.648’,'I70.649’,'I70.65’,'I70.661’,'I70.662’,'I70.663’,'I70.668’,'I70.669’,'I70.691’,'I70.692’,'I70.693’,'I70.698’,'I70.699’,'I70.701’,'I70.702’,'I70.703’,'I70.708’,'I70.709’,'I70.711’,'I70.712’,'I70.713’,'I70.718’,'I70.719’,'I70.721’,'I70.722’,'I70.723’,'I70.728’,'I70.729’,'I70.731’,'I70.732’,'I70.733’,'I70.734’,'I70.735’,'I70.738’,'I70.739’,'I70.741’,'I70.742’,'I70.743’,'I70.744’,'I70.745’,'I70.748’,'I70.749’,'I70.75’,'I70.761’,'I70.762’,'I70.763’,'I70.768’,'I70.769’,'I70.791’,'I70.792’,'I70.793’,'I70.798’,'I70.799’,'I70.8’,'I70.90’,'I70.91’,'I70.92’,'Z95.5’,'Z98.61’, |
| ***Heart Disease*** | '391’,'391.1’,'391.2’,'391.8’,'391.9’,'392’,'392.9’,'393’,'394.1’,'395’,'395.1’,'395.2’,'395.9’,'397.1’,'397.9’,'398’,'398.9’,'398.91’,'398.99’,'401.1’,'401.9’,'402’,'402.01’,'402.1’,'402.11’,'402.9’,'402.91’,'404.01’,'404.02’,'404.03’,'404.1’,'404.11’,'404.12’,'404.13’,'404.9’,'404.91’,'404.92’,'404.93’,'405.01’,'405.09’,'405.11’,'405.19’,'405.91’,'405.99’,'414’,'414’,'414.01’,'414.02’,'414.03’,'414.04’,'414.05’,'414.06’,'414.07’,'414.1’,'414.11’,'414.12’,'414.19’,'414.2’,'414.3’,'414.4’,'414.8’,'414.9’,'415’,'415.1’,'415.11’,'415.12’,'415.13’,'415.19’,'416’,'416.1’,'416.2’,'416.8’,'416.9’,'417’,'417.1’,'417.8’,'417.9’,'424.2’,'424.3’,'428’,'428.1’,'428.2’,'428.21’,'428.22’,'428.23’,'428.3’,'428.31’,'428.32’,'428.33’,'428.4’,'428.41’,'428.42’,'428.43’,'428.9’,'437.3’,'440’,'440.1’,'440.2’,'440.2’,'440.21’,'440.22’,'440.23’,'440.24’,'440.29’,'440.3’,'440.31’,'440.32’,'440.4’,'440.8’,'440.9’,'441.1’,'441.2’,'441.3’,'441.4’,'441.5’,'441.6’,'441.7’,'441.9’,'442’,'442.1’,'442.2’,'442.3’,'442.81’,'442.82’,'442.83’,'442.84’,'442.89’,'442.9’,'459.3’,'459.31’,'459.32’,'459.33’,'459.39’,'517.1’,'714.4’,'725’,'796.2’,'V12.55’,'V82.2’, | 'I00.’,'I01.0’,'I01.1’,'I01.2’,'I01.8’,'I01.9’,'I02.0’,'I02.9’,'I05.0’,'I05.1’,'I05.2’,'I05.8’,'I05.9’,'I06.0’,'I06.1’,'I06.2’,'I06.8’,'I06.9’,'I07.0’,'I07.1’,'I07.2’,'I07.8’,'I07.9’,'I08.0’,'I08.1’,'I08.2’,'I08.3’,'I08.8’,'I08.9’,'I09.0’,'I09.1’,'I09.2’,'I09.81’,'I09.89’,'I09.9’,'I11.0’,'I13.0’,'I13.2’,'I20.0’,'I20.1’,'I20.8’,'I20.9’,'I24.0’,'I24.1’,'I24.8’,'I24.9’,'I25.10’,'I25.110’,'I25.111’,'I25.118’,'I25.119’,'I25.2’,'I25.3’,'I25.41’,'I25.42’,'I25.5’,'I25.6’,'I25.700’,'I25.701’,'I25.708’,'I25.709’,'I25.710’,'I25.711’,'I25.718’,'I25.719’,'I25.720’,'I25.721’,'I25.728’,'I25.729’,'I25.730’,'I25.731’,'I25.738’,'I25.739’,'I25.750’,'I25.751’,'I25.758’,'I25.759’,'I25.760’,'I25.761’,'I25.768’,'I25.769’,'I25.790’,'I25.791’,'I25.798’,'I25.799’,'I25.810’,'I25.811’,'I25.812’,'I25.82’,'I25.83’,'I25.84’,'I25.89’,'I25.9’,'I27.0’,'I27.1’,'I27.2’,'I27.20’,'I27.21’,'I27.22’,'I27.23’,'I27.24’,'I27.29’,'I27.81’,'I27.82’,'I27.83’,'I27.89’,'I27.9’,'I28.0’,'I28.1’,'I28.8’,'I28.9’,'I50.1’,'I50.20’,'I50.21’,'I50.22’,'I50.23’,'I50.30’,'I50.31’,'I50.32’,'I50.33’,'I50.40’,'I50.41’,'I50.42’,'I50.43’,'I50.810’,'I50.811’,'I50.812’,'I50.813’,'I50.814’,'I50.82’,'I50.83’,'I50.84’,'I50.89’,'I50.9’,'I51.0’,'I51.1’,'I51.2’,'I51.3’,'I51.5’,'I51.7’,'I51.81’,'I51.89’,'I51.9’,'I52.’,'I97.130’,'I97.131’,'M05.30’,'M05.311’,'M05.312’,'M05.319’,'M05.321’,'M05.322’,'M05.329’,'M05.331’,'M05.332’,'M05.339’,'M05.341’,'M05.342’,'M05.349’,'M05.351’,'M05.352’,'M05.359’,'M05.361’,'M05.362’,'M05.369’,'M05.371’,'M05.372’,'M05.379’,'M05.39’,'O29.121’,'O29.122’,'O29.123’,'O29.129’,'Z95.1’,'Z95.5’,'Z95.811’,'Z95.812’,'Z98.61’, |
| ***Heart Failure*** | '410.52','410.6','410.6','410.61','410.62','410.7','410.7','410.71','410.72','410.8','410.8','410.81','410.82','410.9','410.9','410.91','410.92','411','411.1','411.8','411.81','411.89','412','414','414','414.01','414.02','414.03','414.04','414.05','414.06','414.07','414.2','414.3','414.4', | 'I09.81','I11.0','I13.0','I13.2','I50.1','I50.20','I50.21','I50.22','I50.23','I50.30','I50.31','I50.32','I50.33','I50.40','I50.41','I50.42','I50.43','I50.810','I50.811','I50.812','I50.813','I50.814','I50.82','I50.83','I50.84','I50.89','I50.9','I97.130','I97.131','O29.121','O29.122','O29.123','O29.129','Z95.811','Z95.812', |
| ***Other Heart Disease*** | '112.81','115.03','115.04','115.13','115.14','115.93','115.94','130.3','364','364.01','364.02','364.03','364.04','364.05','364.1','364.11','364.21','364.22','364.23','364.24','364.3','364.41','364.42','364.51','364.52','364.53','364.54','364.55','364.56','364.57','364.59','364.6','364.61','364.62','364.63','364.64','364.7','364.71','364.72','364.73','364.74','364.75','364.76','364.77','364.8','364.81','364.82','364.89','364.9','391.1','391.2','391.8','391.9','392','392.9','393','394','394.1','394.2','394.9','395.1','395.2','395.9','396.1','396.2','396.3','396.8','396.9','397.1','397.9','398','398.9','398.91','398.99','401.1','401.9','402','402.01','402.1','402.11','402.9','402.91','403.01','403.1','403.11','403.9','403.91','404.01','404.02','404.03','404.1','404.11','404.12','404.13','404.9','404.91','404.92','404.93','405.01','405.09','405.11','405.19','405.91','405.99','410.01','410.02','410.1','410.11','410.12','410.2','410.21','410.22','410.3','410.31','410.32','410.4','410.41','410.42','410.5','410.51','410.52','410.6','410.61','410.62','410.7','410.71','410.72','410.8','410.81','410.82','410.9','410.91','410.92','411.1','411.8','411.81','411.89','412','413','413.1','413.9','414.01','414.06','414.11','414.12','414.19','414.2','414.3','414.4','414.8','414.9','415.1','415.12','415.13','415.19','416.1','416.2','416.8','416.9','417.1','417.8','417.9','420','420.9','420.91','420.99','421','421.1','421.9','422','422.9','422.91','422.92','422.93','422.99','423','423.1','423.2','423.3','423.8','423.9','424','424.1','424.2','424.3','424.9','424.91','424.99','425','425.1','425.11','425.18','425.2','425.3','425.4','425.7','425.8','425.9','426','426.1','426.11','426.12','426.13','426.2','426.3','426.4','426.5','426.51','426.52','426.53','426.54','426.6','426.7','426.81','426.82','426.89','426.9','427','427.1','427.2','427.31','427.32','427.41','427.42','427.5','427.6','427.61','427.69','427.81','427.89','427.9','428.1','428.21','428.22','428.23','428.31','428.32','428.33','428.41','428.42','428.43','428.9','429','429.1','429.2','429.3','429.4','429.5','429.6','429.71','429.79','429.81','429.82','429.83','429.89','429.9','437.2','742.1','742.2','742.3','785.1','785.2','785.3','786.51','786.59','V12.55','V42.2','V43.3','V45.0','V45.00','V45.01','V45.02','V45.09','V45.81','V45.82','V53.3','V53.31','V53.32','V53.39', | 'I25.3','I25.41','I51.0','I51.1','I51.2','I51.3','I51.5','I51.7','I51.81','I51.89','I51.9','I52.','M05.30','M05.311','M05.312','M05.319','M05.321','M05.322','M05.329','M05.331','M05.332','M05.339','M05.341','M05.342','M05.349','M05.351','M05.352','M05.359','M05.361','M05.362','M05.369','M05.371','M05.372','M05.379','M05.39', |
| ***Peripheral Arterial Disease*** | '250.71','250.72','250.73','440.2','440.21','440.22','440.23','440.24','440.29','440.31','440.32','440.4','440.9','443.9','444.22','444.81','444.89','445.02','447.1','459.9','707.1','707.11','707.12','707.13','707.14','707.15','707.19','785.4', | 'E08.52','E09.52','E10.51','E10.52','E10.59','E11.51','E11.52','E11.59','E13.51','E13.52','E13.59','I70.0','I70.201','I70.202','I70.203','I70.208','I70.209','I70.211','I70.212','I70.213','I70.218','I70.219','I70.221','I70.222','I70.223','I70.228','I70.229','I70.231','I70.232','I70.233','I70.234','I70.235','I70.238','I70.239','I70.241','I70.242','I70.243','I70.244','I70.245','I70.248','I70.249','I70.25','I70.261','I70.262','I70.263','I70.268','I70.269','I70.291','I70.292','I70.293','I70.298','I70.299','I70.301','I70.302','I70.303','I70.308','I70.309','I70.311','I70.312','I70.313','I70.318','I70.319','I70.321','I70.322','I70.323','I70.328','I70.329','I70.331','I70.332','I70.333','I70.334','I70.335','I70.338','I70.339','I70.341','I70.342','I70.343','I70.344','I70.345','I70.348','I70.349','I70.35','I70.361','I70.362','I70.363','I70.368','I70.369','I70.391','I70.392','I70.393','I70.398','I70.399','I70.401','I70.402','I70.403','I70.408','I70.409','I70.411','I70.412','I70.413','I70.418','I70.419','I70.421','I70.422','I70.423','I70.428','I70.429','I70.431','I70.432','I70.433','I70.434','I70.435','I70.438','I70.439','I70.441','I70.442','I70.443','I70.444','I70.445','I70.448','I70.449','I70.45','I70.461','I70.462','I70.463','I70.468','I70.469','I70.491','I70.492','I70.493','I70.498','I70.499','I70.501','I70.502','I70.503','I70.508','I70.509','I70.511','I70.512','I70.513','I70.518','I70.519','I70.521','I70.522','I70.523','I70.528','I70.529','I70.531','I70.532','I70.533','I70.534','I70.535','I70.538','I70.539','I70.541','I70.542','I70.543','I70.544','I70.545','I70.548','I70.549','I70.55','I70.561','I70.562','I70.563','I70.568','I70.569','I70.591','I70.592','I70.593','I70.598','I70.599','I70.601','I70.602','I70.603','I70.608','I70.609','I70.611','I70.612','I70.613','I70.618','I70.619','I70.621','I70.622','I70.623','I70.628','I70.629','I70.631','I70.632','I70.633','I70.634','I70.635','I70.638','I70.639','I70.641','I70.642','I70.643','I70.644','I70.645','I70.648','I70.649','I70.65','I70.661','I70.662','I70.663','I70.668','I70.669','I70.691','I70.692','I70.693','I70.698','I70.699','I70.701','I70.702','I70.703','I70.708','I70.709','I70.711','I70.712','I70.713','I70.718','I70.719','I70.721','I70.722','I70.723','I70.728','I70.729','I70.731','I70.732','I70.733','I70.734','I70.735','I70.738','I70.739','I70.741','I70.742','I70.743','I70.744','I70.745','I70.748','I70.749','I70.75','I70.761','I70.762','I70.763','I70.768','I70.769','I70.791','I70.792','I70.793','I70.798','I70.799','I70.90','I70.91','I70.92','I73.9','I74.01','I74.09','I74.10','I74.19','I74.3','I74.4','I74.5','I74.8','I75.021','I75.022','I75.023','I75.029','I77.1','I96.','L97.101','L97.102','L97.103','L97.104','L97.109','L97.111','L97.112','L97.113','L97.114','L97.119','L97.121','L97.122','L97.123','L97.124','L97.129','L97.201','L97.202','L97.203','L97.204','L97.209','L97.211','L97.212','L97.213','L97.214','L97.219','L97.221','L97.222','L97.223','L97.224','L97.229','L97.301','L97.302','L97.303','L97.304','L97.309','L97.311','L97.312','L97.313','L97.314','L97.319','L97.321','L97.322','L97.323','L97.324','L97.329','L97.401','L97.402','L97.403','L97.404','L97.409','L97.411','L97.412','L97.413','L97.414','L97.419','L97.421','L97.422','L97.423','L97.424','L97.429','L97.501','L97.502','L97.503','L97.504','L97.509','L97.511','L97.512','L97.513','L97.514','L97.519','L97.521','L97.522','L97.523','L97.524','L97.529','L97.801','L97.802','L97.803','L97.804','L97.809','L97.811','L97.812','L97.813','L97.814','L97.819','L97.821','L97.822','L97.823','L97.824','L97.829','L97.901','L97.902','L97.903','L97.904','L97.909','L97.911','L97.912','L97.913','L97.914','L97.919','L97.921','L97.922','L97.923','L97.924','L97.929', |
| **Metabolic Disorders** | | |
| ***Any Diabetes*** | '249','249.01','249.1','249.11','249.2','249.21','249.3','249.31','249.4','249.41','249.5','249.51','249.6','249.61','249.7','249.71','249.8','249.81','249.9','249.91','250','250.01','250.02','250.03','250.1','250.11','250.12','250.13','250.2','250.21','250.22','250.23','250.3','250.31','250.32','250.33','250.4','250.41','250.42','250.43','250.5','250.51','250.52','250.53','250.6','250.61','250.62','250.63','250.7','250.71','250.72','250.73','250.8','250.81','250.82','250.83','250.9','250.91','250.92','250.93','253.5','357.2','648.01','648.02','648.03','648.04','775.1','V18.0','588.1','V12.21','V77.1','250','250.02','250.1','250.12','250.2','250.22','250.3','250.32','250.4','250.42','250.5','250.52','250.6','250.62','250.7','250.72','250.8','250.82','250.9','250.92','357.2','362.01','362.02','362.03','362.04','362.05','362.06','362.07','366.41','648','648.01','648.02','648.03','648.04','249.1','249.2','249.3','249.4','249.5','249.6','249.7','249.8','249.9','250.1','250.11','250.2','250.3','250.31','250.4','250.41','250.5','250.51','250.6','250.61','250.7','250.71','250.8','250.81','250.9','250.91','251.1','357.2','362.01','362.02','362.04','362.05','362.06','362.07','366.41','249','250','250.01','251.3','648','648.01','648.02','648.03','648.04','648.81','648.82','648.83','648.84','272','272.1','272.2','272.3','272.4','272','272.1','272.2','272.3','272.4','272.5','272.6','272.7','272.8','272.9','286.53','330.1','330.2','362.71','414.3', | 'E08.00','E08.01','E08.10','E08.11','E08.21','E08.22','E08.29','E08.311','E08.319','E08.321','E08.3211','E08.3212','E08.3213','E08.3219','E08.329','E08.3291','E08.3292','E08.3293','E08.3299','E08.331','E08.3311','E08.3312','E08.3313','E08.3319','E08.339','E08.3391','E08.3392','E08.3393','E08.3399','E08.341','E08.3411','E08.3412','E08.3413','E08.3419','E08.349','E08.3491','E08.3492','E08.3493','E08.3499','E08.351','E08.3511','E08.3512','E08.3513','E08.3519','E08.3521','E08.3522','E08.3523','E08.3529','E08.3531','E08.3532','E08.3533','E08.3539','E08.3541','E08.3542','E08.3543','E08.3549','E08.3551','E08.3552','E08.3553','E08.3559','E08.359','E08.3591','E08.3592','E08.3593','E08.3599','E08.36','E08.37X1','E08.37X2','E08.37X3','E08.37X9','E08.39','E08.40','E08.41','E08.42','E08.43','E08.44','E08.49','E08.51','E08.52','E08.59','E08.610','E08.618','E08.620','E08.621','E08.622','E08.628','E08.630','E08.638','E08.641','E08.649','E08.65','E08.69','E08.8','E08.9','E09.00','E09.01','E09.10','E09.11','E09.21','E09.22','E09.29','E09.311','E09.319','E09.321','E09.3211','E09.3212','E09.3213','E09.3219','E09.329','E09.3291','E09.3292','E09.3293','E09.3299','E09.331','E09.3311','E09.3312','E09.3313','E09.3319','E09.339','E09.3391','E09.3392','E09.3393','E09.3399','E09.341','E09.3411','E09.3412','E09.3413','E09.3419','E09.349','E09.3491','E09.3492','E09.3493','E09.3499','E09.351','E09.3511','E09.3512','E09.3513','E09.3519','E09.3521','E09.3522','E09.3523','E09.3529','E09.3531','E09.3532','E09.3533','E09.3539','E09.3541','E09.3542','E09.3543','E09.3549','E09.3551','E09.3552','E09.3553','E09.3559','E09.359','E09.3591','E09.3592','E09.3593','E09.3599','E09.36','E09.37X1','E09.37X2','E09.37X3','E09.37X9','E09.39','E09.40','E09.41','E09.42','E09.43','E09.44','E09.49','E09.51','E09.52','E09.59','E09.610','E09.618','E09.620','E09.621','E09.622','E09.628','E09.630','E09.638','E09.641','E09.649','E09.65','E09.69','E09.8','E09.9','E10.10','E10.11','E10.21','E10.22','E10.29','E10.311','E10.319','E10.321','E10.3211','E10.3212','E10.3213','E10.3219','E10.329','E10.3291','E10.3292','E10.3293','E10.3299','E10.331','E10.3311','E10.3312','E10.3313','E10.3319','E10.339','E10.3391','E10.3392','E10.3393','E10.3399','E10.341','E10.3411','E10.3412','E10.3413','E10.3419','E10.349','E10.3491','E10.3492','E10.3493','E10.3499','E10.351','E10.3511','E10.3512','E10.3513','E10.3519','E10.3521','E10.3522','E10.3523','E10.3529','E10.3531','E10.3532','E10.3533','E10.3539','E10.3541','E10.3542','E10.3543','E10.3549','E10.3551','E10.3552','E10.3553','E10.3559','E10.359','E10.3591','E10.3592','E10.3593','E10.3599','E10.36','E10.37X1','E10.37X2','E10.37X3','E10.37X9','E10.39','E10.40','E10.41','E10.42','E10.43','E10.44','E10.49','E10.51','E10.52','E10.59','E10.610','E10.618','E10.620','E10.621','E10.622','E10.628','E10.630','E10.638','E10.641','E10.649','E10.65','E10.69','E10.8','E10.9','E11.00','E11.01','E11.10','E11.11','E11.21','E11.22','E11.29','E11.311','E11.319','E11.321','E11.3211','E11.3212','E11.3213','E11.3219','E11.329','E11.3291','E11.3292','E11.3293','E11.3299','E11.331','E11.3311','E11.3312','E11.3313','E11.3319','E11.339','E11.3391','E11.3392','E11.3393','E11.3399','E11.341','E11.3411','E11.3412','E11.3413','E11.3419','E11.349','E11.3491','E11.3492','E11.3493','E11.3499','E11.351','E11.3511','E11.3512','E11.3513','E11.3519','E11.3521','E11.3522','E11.3523','E11.3529','E11.3531','E11.3532','E11.3533','E11.3539','E11.3541','E11.3542','E11.3543','E11.3549','E11.3551','E11.3552','E11.3553','E11.3559','E11.359','E11.3591','E11.3592','E11.3593','E11.3599','E11.36','E11.37X1','E11.37X2','E11.37X3','E11.37X9','E11.39','E11.40','E11.41','E11.42','E11.43','E11.44','E11.49','E11.51','E11.52','E11.59','E11.610','E11.618','E11.620','E11.621','E11.622','E11.628','E11.630','E11.638','E11.641','E11.649','E11.65','E11.69','E11.8','E11.9','E13.00','E13.01','E13.10','E13.11','E13.21','E13.22','E13.29','E13.311','E13.319','E13.321','E13.3211','E13.3212','E13.3213','E13.3219','E13.329','E13.3291','E13.3292','E13.3293','E13.3299','E13.331','E13.3311','E13.3312','E13.3313','E13.3319','E13.339','E13.3391','E13.3392','E13.3393','E13.3399','E13.341','E13.3411','E13.3412','E13.3413','E13.3419','E13.349','E13.3491','E13.3492','E13.3493','E13.3499','E13.351','E13.3511','E13.3512','E13.3513','E13.3519','E13.3521','E13.3522','E13.3523','E13.3529','E13.3531','E13.3532','E13.3533','E13.3539','E13.3541','E13.3542','E13.3543','E13.3549','E13.3551','E13.3552','E13.3553','E13.3559','E13.359','E13.3591','E13.3592','E13.3593','E13.3599','E13.36','E13.37X1','E13.37X2','E13.37X3','E13.37X9','E13.39','E13.40','E13.41','E13.42','E13.43','E13.44','E13.49','E13.51','E13.52','E13.59','E13.610','E13.618','E13.620','E13.621','E13.622','E13.628','E13.630','E13.638','E13.641','E13.649','E13.65','E13.69','E13.8','E13.9','E89.1','O24.011','O24.012','O24.013','O24.019','O24.02','O24.03','O24.111','O24.112','O24.113','O24.119','O24.12','O24.13','O24.311','O24.312','O24.313','O24.319','O24.32','O24.33','O24.410','O24.414','O24.415','O24.419','O24.420','O24.424','O24.425','O24.429','O24.430','O24.434','O24.435','O24.439','O24.811','O24.812','O24.813','O24.819','O24.82','O24.83','O24.911','O24.912','O24.913','O24.919','O24.92','O24.93','O99.810','O99.814','O99.815','E08.00','E08.01','E08.10','E08.11','E08.21','E08.22','E08.29','E08.311','E08.319','E08.321','E08.3211','E08.3212','E08.3213','E08.3219','E08.329','E08.3291','E08.3292','E08.3293','E08.3299','E08.331','E08.3311','E08.3312','E08.3313','E08.3319','E08.339','E08.3391','E08.3392','E08.3393','E08.3399','E08.341','E08.3411','E08.3412','E08.3413','E08.3419','E08.349','E08.3491','E08.3492','E08.3493','E08.3499','E08.351','E08.3511','E08.3512','E08.3513','E08.3519','E08.3521','E08.3522','E08.3523','E08.3529','E08.3531','E08.3532','E08.3533','E08.3539','E08.3541','E08.3542','E08.3543','E08.3549','E08.3551','E08.3552','E08.3553','E08.3559','E08.359','E08.3591','E08.3592','E08.3593','E08.3599','E08.36','E08.37X1','E08.37X2','E08.37X3','E08.37X9','E08.39','E08.40','E08.41','E08.42','E08.43','E08.44','E08.49','E08.51','E08.52','E08.59','E08.610','E08.618','E08.620','E08.621','E08.622','E08.628','E08.630','E08.638','E08.641','E08.649','E08.65','E08.69','E08.8','E08.9','E09.00','E09.01','E09.10','E09.11','E09.21','E09.22','E09.29','E09.311','E09.319','E09.321','E09.3211','E09.3212','E09.3213','E09.3219','E09.329','E09.3291','E09.3292','E09.3293','E09.3299','E09.331','E09.3311','E09.3312','E09.3313','E09.3319','E09.339','E09.3391','E09.3392','E09.3393','E09.3399','E09.341','E09.3411','E09.3412','E09.3413','E09.3419','E09.349','E09.3491','E09.3492','E09.3493','E09.3499','E09.351','E09.3511','E09.3512','E09.3513','E09.3519','E09.3521','E09.3522','E09.3523','E09.3529','E09.3531','E09.3532','E09.3533','E09.3539','E09.3541','E09.3542','E09.3543','E09.3549','E09.3551','E09.3552','E09.3553','E09.3559','E09.359','E09.3591','E09.3592','E09.3593','E09.3599','E09.36','E09.37X1','E09.37X2','E09.37X3','E09.37X9','E09.39','E09.40','E09.41','E09.42','E09.43','E09.44','E09.49','E09.51','E09.52','E09.59','E09.610','E09.618','E09.620','E09.621','E09.622','E09.628','E09.630','E09.638','E09.641','E09.649','E09.65','E09.69','E09.8','E09.9','E13.00','E13.01','E13.10','E13.11','E13.21','E13.22','E13.29','E13.311','E13.319','E13.321','E13.3211','E13.3212','E13.3213','E13.3219','E13.329','E13.3291','E13.3292','E13.3293','E13.3299','E13.331','E13.3311','E13.3312','E13.3313','E13.3319','E13.339','E13.3391','E13.3392','E13.3393','E13.3399','E13.341','E13.3411','E13.3412','E13.3413','E13.3419','E13.349','E13.3491','E13.3492','E13.3493','E13.3499','E13.351','E13.3511','E13.3512','E13.3513','E13.3519','E13.3521','E13.3522','E13.3523','E13.3529','E13.3531','E13.3532','E13.3533','E13.3539','E13.3541','E13.3542','E13.3543','E13.3549','E13.3551','E13.3552','E13.3553','E13.3559','E13.359','E13.3591','E13.3592','E13.3593','E13.3599','E13.36','E13.39','E13.40','E13.41','E13.42','E13.43','E13.44','E13.49','E13.51','E13.52','E13.59','E13.610','E13.618','E13.620','E13.621','E13.622','E13.628','E13.630','E13.638','E13.641','E13.649','E13.65','E13.69','E13.8','E13.9','E11.00','E11.01','E11.10','E11.11','E11.21','E11.22','E11.29','E11.311','E11.319','E11.321','E11.3211','E11.3212','E11.3213','E11.3219','E11.329','E11.3291','E11.3292','E11.3293','E11.3299','E11.331','E11.3311','E11.3312','E11.3313','E11.3319','E11.339','E11.3391','E11.3392','E11.3393','E11.3399','E11.341','E11.3411','E11.3412','E11.3413','E11.3419','E11.349','E11.3491','E11.3492','E11.3493','E11.3499','E11.351','E11.3511','E11.3512','E11.3513','E11.3519','E11.3521','E11.3522','E11.3523','E11.3529','E11.3531','E11.3532','E11.3533','E11.3539','E11.3541','E11.3542','E11.3543','E11.3549','E11.3551','E11.3552','E11.3553','E11.3559','E11.359','E11.3591','E11.3592','E11.3593','E11.3599','E11.36','E11.37X1','E11.37X2','E11.37X3','E11.37X9','E11.39','E11.40','E11.41','E11.42','E11.43','E11.44','E11.49','E11.51','E11.52','E11.59','E11.610','E11.618','E11.620','E11.621','E11.622','E11.628','E11.630','E11.638','E11.641','E11.649','E11.65','E11.69','E11.8','E11.9','O24.111','O24.112','O24.113','O24.119','O24.12','O24.13','E08.00','E08.01','E08.10','E08.11','E08.21','E08.22','E08.29','E08.311','E08.319','E08.321','E08.3211','E08.3212','E08.3213','E08.3219','E08.329','E08.3291','E08.3292','E08.3293','E08.3299','E08.331','E08.3311','E08.3312','E08.3313','E08.3319','E08.339','E08.3391','E08.3392','E08.3393','E08.3399','E08.341','E08.3411','E08.3412','E08.3413','E08.3419','E08.349','E08.3491','E08.3492','E08.3493','E08.3499','E08.351','E08.3511','E08.3512','E08.3513','E08.3519','E08.3521','E08.3522','E08.3523','E08.3529','E08.3531','E08.3532','E08.3533','E08.3539','E08.3541','E08.3542','E08.3543','E08.3549','E08.3551','E08.3552','E08.3553','E08.3559','E08.359','E08.3591','E08.3592','E08.3593','E08.3599','E08.36','E08.37X1','E08.37X2','E08.37X3','E08.37X9','E08.39','E08.40','E08.41','E08.42','E08.43','E08.44','E08.49','E08.51','E08.52','E08.59','E08.610','E08.618','E08.620','E08.621','E08.622','E08.628','E08.630','E08.638','E08.641','E08.649','E08.65','E08.69','E08.8','E09.00','E09.01','E09.10','E09.11','E09.21','E09.22','E09.29','E09.311','E09.319','E09.321','E09.3211','E09.3212','E09.3213','E09.3219','E09.329','E09.3291','E09.3292','E09.3293','E09.3299','E09.331','E09.3311','E09.3312','E09.3313','E09.3319','E09.339','E09.3391','E09.3392','E09.3393','E09.3399','E09.341','E09.3411','E09.3412','E09.3413','E09.3419','E09.349','E09.3491','E09.3492','E09.3493','E09.3499','E09.351','E09.3511','E09.3512','E09.3513','E09.3519','E09.3521','E09.3522','E09.3523','E09.3529','E09.3531','E09.3532','E09.3533','E09.3539','E09.3541','E09.3542','E09.3543','E09.3549','E09.3551','E09.3552','E09.3553','E09.3559','E09.359','E09.3591','E09.3592','E09.3593','E09.3599','E09.36','E09.37X1','E09.37X2','E09.37X3','E09.37X9','E09.39','E09.40','E09.41','E09.42','E09.43','E09.44','E09.49','E09.51','E09.52','E09.59','E09.610','E09.618','E09.620','E09.621','E09.622','E09.628','E09.630','E09.638','E09.641','E09.649','E09.65','E09.69','E09.8','E10.10','E10.11','E10.21','E10.22','E10.29','E10.311','E10.319','E10.321','E10.3211','E10.3212','E10.3213','E10.3219','E10.329','E10.3291','E10.3292','E10.3293','E10.3299','E10.331','E10.3311','E10.3312','E10.3313','E10.3319','E10.339','E10.3391','E10.3392','E10.3393','E10.3399','E10.341','E10.3411','E10.3412','E10.3413','E10.3419','E10.349','E10.3491','E10.3492','E10.3493','E10.3499','E10.351','E10.3511','E10.3512','E10.3513','E10.3519','E10.3521','E10.3522','E10.3523','E10.3529','E10.3531','E10.3532','E10.3533','E10.3539','E10.3541','E10.3542','E10.3543','E10.3549','E10.3551','E10.3552','E10.3553','E10.3559','E10.359','E10.3591','E10.3592','E10.3593','E10.3599','E10.36','E10.37X1','E10.37X2','E10.37X3','E10.37X9','E10.39','E10.40','E10.41','E10.42','E10.43','E10.44','E10.49','E10.51','E10.52','E10.59','E10.610','E10.618','E10.620','E10.621','E10.622','E10.628','E10.630','E10.638','E10.641','E10.649','E10.65','E10.69','E10.8','E11.00','E11.01','E11.10','E11.11','E11.21','E11.22','E11.29','E11.311','E11.319','E11.321','E11.3211','E11.3212','E11.3213','E11.3219','E11.329','E11.3291','E11.3292','E11.3293','E11.3299','E11.331','E11.3311','E11.3312','E11.3313','E11.3319','E11.339','E11.3391','E11.3392','E11.3393','E11.3399','E11.341','E11.3411','E11.3412','E11.3413','E11.3419','E11.349','E11.3491','E11.3492','E11.3493','E11.3499','E11.351','E11.3511','E11.3512','E11.3513','E11.3519','E11.3521','E11.3522','E11.3523','E11.3529','E11.3531','E11.3532','E11.3533','E11.3539','E11.3541','E11.3542','E11.3543','E11.3549','E11.3551','E11.3552','E11.3553','E11.3559','E11.359','E11.3591','E11.3592','E11.3593','E11.3599','E11.36','E11.37X1','E11.37X2','E11.37X3','E11.37X9','E11.39','E11.40','E11.41','E11.42','E11.43','E11.44','E11.49','E11.51','E11.52','E11.59','E11.610','E11.618','E11.620','E11.621','E11.622','E11.628','E11.630','E11.638','E11.641','E11.649','E11.65','E11.69','E11.8','E13.00','E13.01','E13.10','E13.11','E13.21','E13.22','E13.29','E13.311','E13.319','E13.321','E13.3211','E13.3212','E13.3213','E13.3219','E13.329','E13.3291','E13.3292','E13.3293','E13.3299','E13.331','E13.3311','E13.3312','E13.3313','E13.3319','E13.339','E13.3391','E13.3392','E13.3393','E13.3399','E13.341','E13.3411','E13.3412','E13.3413','E13.3419','E13.349','E13.3491','E13.3492','E13.3493','E13.3499','E13.351','E13.3511','E13.3512','E13.3513','E13.3519','E13.3521','E13.3522','E13.3523','E13.3529','E13.3531','E13.3532','E13.3533','E13.3539','E13.3541','E13.3542','E13.3543','E13.3549','E13.3551','E13.3552','E13.3553','E13.3559','E13.359','E13.3591','E13.3592','E13.3593','E13.3599','E13.36','E13.37X1','E13.37X2','E13.37X3','E13.37X9','E13.39','E13.40','E13.41','E13.42','E13.43','E13.44','E13.49','E13.51','E13.52','E13.59','E13.610','E13.618','E13.620','E13.621','E13.622','E13.628','E13.630','E13.638','E13.641','E13.649','E13.65','E13.69','E13.8','E08.9','E09.9','E10.9','E11.9','E13.9','E89.1','O24.011','O24.012','O24.013','O24.019','O24.02','O24.03','O24.111','O24.112','O24.113','O24.119','O24.12','O24.13','O24.311','O24.312','O24.313','O24.319','O24.32','O24.33','O24.410','O24.414','O24.415','O24.419','O24.420','O24.424','O24.425','O24.429','O24.430','O24.434','O24.435','O24.439','O24.811','O24.812','O24.813','O24.819','O24.82','O24.83','O24.911','O24.912','O24.913','O24.919','O24.92','O24.93','O99.810','O99.814','O99.815','E78.0','E78.00','E78.01','E78.1','E78.2','E78.3','E78.4','E78.41','E78.49','E78.5','D68.312','E75.00','E75.01','E75.02','E75.09','E75.10','E75.11','E75.19','E75.21','E75.22','E75.23','E75.240','E75.241','E75.242','E75.243','E75.248','E75.249','E75.25','E75.26','E75.29','E75.3','E75.4','E75.5','E75.6','E78.0','E78.00','E78.01','E78.1','E78.2','E78.3','E78.4','E78.41','E78.49','E78.5','E78.6','E78.70','E78.71','E78.72','E78.79','E78.81','E78.89','E78.9','I25.83', |
| ***Other Diabetes*** | '588.1','V12.21','V77.1', | 'E08.00','E08.01','E08.10','E08.11','E08.21','E08.22','E08.29','E08.311','E08.319','E08.321','E08.3211','E08.3212','E08.3213','E08.3219','E08.329','E08.3291','E08.3292','E08.3293','E08.3299','E08.331','E08.3311','E08.3312','E08.3313','E08.3319','E08.339','E08.3391','E08.3392','E08.3393','E08.3399','E08.341','E08.3411','E08.3412','E08.3413','E08.3419','E08.349','E08.3491','E08.3492','E08.3493','E08.3499','E08.351','E08.3511','E08.3512','E08.3513','E08.3519','E08.3521','E08.3522','E08.3523','E08.3529','E08.3531','E08.3532','E08.3533','E08.3539','E08.3541','E08.3542','E08.3543','E08.3549','E08.3551','E08.3552','E08.3553','E08.3559','E08.359','E08.3591','E08.3592','E08.3593','E08.3599','E08.36','E08.37X1','E08.37X2','E08.37X3','E08.37X9','E08.39','E08.40','E08.41','E08.42','E08.43','E08.44','E08.49','E08.51','E08.52','E08.59','E08.610','E08.618','E08.620','E08.621','E08.622','E08.628','E08.630','E08.638','E08.641','E08.649','E08.65','E08.69','E08.8','E08.9','E09.00','E09.01','E09.10','E09.11','E09.21','E09.22','E09.29','E09.311','E09.319','E09.321','E09.3211','E09.3212','E09.3213','E09.3219','E09.329','E09.3291','E09.3292','E09.3293','E09.3299','E09.331','E09.3311','E09.3312','E09.3313','E09.3319','E09.339','E09.3391','E09.3392','E09.3393','E09.3399','E09.341','E09.3411','E09.3412','E09.3413','E09.3419','E09.349','E09.3491','E09.3492','E09.3493','E09.3499','E09.351','E09.3511','E09.3512','E09.3513','E09.3519','E09.3521','E09.3522','E09.3523','E09.3529','E09.3531','E09.3532','E09.3533','E09.3539','E09.3541','E09.3542','E09.3543','E09.3549','E09.3551','E09.3552','E09.3553','E09.3559','E09.359','E09.3591','E09.3592','E09.3593','E09.3599','E09.36','E09.37X1','E09.37X2','E09.37X3','E09.37X9','E09.39','E09.40','E09.41','E09.42','E09.43','E09.44','E09.49','E09.51','E09.52','E09.59','E09.610','E09.618','E09.620','E09.621','E09.622','E09.628','E09.630','E09.638','E09.641','E09.649','E09.65','E09.69','E09.8','E09.9','E13.00','E13.01','E13.10','E13.11','E13.21','E13.22','E13.29','E13.311','E13.319','E13.321','E13.3211','E13.3212','E13.3213','E13.3219','E13.329','E13.3291','E13.3292','E13.3293','E13.3299','E13.331','E13.3311','E13.3312','E13.3313','E13.3319','E13.339','E13.3391','E13.3392','E13.3393','E13.3399','E13.341','E13.3411','E13.3412','E13.3413','E13.3419','E13.349','E13.3491','E13.3492','E13.3493','E13.3499','E13.351','E13.3511','E13.3512','E13.3513','E13.3519','E13.3521','E13.3522','E13.3523','E13.3529','E13.3531','E13.3532','E13.3533','E13.3539','E13.3541','E13.3542','E13.3543','E13.3549','E13.3551','E13.3552','E13.3553','E13.3559','E13.359','E13.3591','E13.3592','E13.3593','E13.3599','E13.36','E13.39','E13.40','E13.41','E13.42','E13.43','E13.44','E13.49','E13.51','E13.52','E13.59','E13.610','E13.618','E13.620','E13.621','E13.622','E13.628','E13.630','E13.638','E13.641','E13.649','E13.65','E13.69','E13.8','E13.9', |
| ***Diabetes Type Two*** | '250','250.02','250.1','250.12','250.2','250.22','250.3','250.32','250.4','250.42','250.5','250.52','250.6','250.62','250.7','250.72','250.8','250.82','250.9','250.92','357.2','362.01','362.02','362.03','362.04','362.05','362.06','362.07','366.41','648','648.01','648.02','648.03','648.04', | 'E11.00','E11.01','E11.10','E11.11','E11.21','E11.22','E11.29','E11.311','E11.319','E11.321','E11.3211','E11.3212','E11.3213','E11.3219','E11.329','E11.3291','E11.3292','E11.3293','E11.3299','E11.331','E11.3311','E11.3312','E11.3313','E11.3319','E11.339','E11.3391','E11.3392','E11.3393','E11.3399','E11.341','E11.3411','E11.3412','E11.3413','E11.3419','E11.349','E11.3491','E11.3492','E11.3493','E11.3499','E11.351','E11.3511','E11.3512','E11.3513','E11.3519','E11.3521','E11.3522','E11.3523','E11.3529','E11.3531','E11.3532','E11.3533','E11.3539','E11.3541','E11.3542','E11.3543','E11.3549','E11.3551','E11.3552','E11.3553','E11.3559','E11.359','E11.3591','E11.3592','E11.3593','E11.3599','E11.36','E11.37X1','E11.37X2','E11.37X3','E11.37X9','E11.39','E11.40','E11.41','E11.42','E11.43','E11.44','E11.49','E11.51','E11.52','E11.59','E11.610','E11.618','E11.620','E11.621','E11.622','E11.628','E11.630','E11.638','E11.641','E11.649','E11.65','E11.69','E11.8','E11.9','O24.111','O24.112','O24.113','O24.119','O24.12','O24.13', |
| ***Diabetes With Complications*** | '249.1','249.2','249.3','249.4','249.5','249.6','249.7','249.8','249.9','250.1','250.11','250.2','250.3','250.31','250.4','250.41','250.5','250.51','250.6','250.61','250.7','250.71','250.8','250.81','250.9','250.91','251.1','357.2','362.01','362.02','362.04','362.05','362.06','362.07','366.41', | 'E08.00','E08.01','E08.10','E08.11','E08.21','E08.22','E08.29','E08.311','E08.319','E08.321','E08.3211','E08.3212','E08.3213','E08.3219','E08.329','E08.3291','E08.3292','E08.3293','E08.3299','E08.331','E08.3311','E08.3312','E08.3313','E08.3319','E08.339','E08.3391','E08.3392','E08.3393','E08.3399','E08.341','E08.3411','E08.3412','E08.3413','E08.3419','E08.349','E08.3491','E08.3492','E08.3493','E08.3499','E08.351','E08.3511','E08.3512','E08.3513','E08.3519','E08.3521','E08.3522','E08.3523','E08.3529','E08.3531','E08.3532','E08.3533','E08.3539','E08.3541','E08.3542','E08.3543','E08.3549','E08.3551','E08.3552','E08.3553','E08.3559','E08.359','E08.3591','E08.3592','E08.3593','E08.3599','E08.36','E08.37X1','E08.37X2','E08.37X3','E08.37X9','E08.39','E08.40','E08.41','E08.42','E08.43','E08.44','E08.49','E08.51','E08.52','E08.59','E08.610','E08.618','E08.620','E08.621','E08.622','E08.628','E08.630','E08.638','E08.641','E08.649','E08.65','E08.69','E08.8','E09.00','E09.01','E09.10','E09.11','E09.21','E09.22','E09.29','E09.311','E09.319','E09.321','E09.3211','E09.3212','E09.3213','E09.3219','E09.329','E09.3291','E09.3292','E09.3293','E09.3299','E09.331','E09.3311','E09.3312','E09.3313','E09.3319','E09.339','E09.3391','E09.3392','E09.3393','E09.3399','E09.341','E09.3411','E09.3412','E09.3413','E09.3419','E09.349','E09.3491','E09.3492','E09.3493','E09.3499','E09.351','E09.3511','E09.3512','E09.3513','E09.3519','E09.3521','E09.3522','E09.3523','E09.3529','E09.3531','E09.3532','E09.3533','E09.3539','E09.3541','E09.3542','E09.3543','E09.3549','E09.3551','E09.3552','E09.3553','E09.3559','E09.359','E09.3591','E09.3592','E09.3593','E09.3599','E09.36','E09.37X1','E09.37X2','E09.37X3','E09.37X9','E09.39','E09.40','E09.41','E09.42','E09.43','E09.44','E09.49','E09.51','E09.52','E09.59','E09.610','E09.618','E09.620','E09.621','E09.622','E09.628','E09.630','E09.638','E09.641','E09.649','E09.65','E09.69','E09.8','E10.10','E10.11','E10.21','E10.22','E10.29','E10.311','E10.319','E10.321','E10.3211','E10.3212','E10.3213','E10.3219','E10.329','E10.3291','E10.3292','E10.3293','E10.3299','E10.331','E10.3311','E10.3312','E10.3313','E10.3319','E10.339','E10.3391','E10.3392','E10.3393','E10.3399','E10.341','E10.3411','E10.3412','E10.3413','E10.3419','E10.349','E10.3491','E10.3492','E10.3493','E10.3499','E10.351','E10.3511','E10.3512','E10.3513','E10.3519','E10.3521','E10.3522','E10.3523','E10.3529','E10.3531','E10.3532','E10.3533','E10.3539','E10.3541','E10.3542','E10.3543','E10.3549','E10.3551','E10.3552','E10.3553','E10.3559','E10.359','E10.3591','E10.3592','E10.3593','E10.3599','E10.36','E10.37X1','E10.37X2','E10.37X3','E10.37X9','E10.39','E10.40','E10.41','E10.42','E10.43','E10.44','E10.49','E10.51','E10.52','E10.59','E10.610','E10.618','E10.620','E10.621','E10.622','E10.628','E10.630','E10.638','E10.641','E10.649','E10.65','E10.69','E10.8','E11.00','E11.01','E11.10','E11.11','E11.21','E11.22','E11.29','E11.311','E11.319','E11.321','E11.3211','E11.3212','E11.3213','E11.3219','E11.329','E11.3291','E11.3292','E11.3293','E11.3299','E11.331','E11.3311','E11.3312','E11.3313','E11.3319','E11.339','E11.3391','E11.3392','E11.3393','E11.3399','E11.341','E11.3411','E11.3412','E11.3413','E11.3419','E11.349','E11.3491','E11.3492','E11.3493','E11.3499','E11.351','E11.3511','E11.3512','E11.3513','E11.3519','E11.3521','E11.3522','E11.3523','E11.3529','E11.3531','E11.3532','E11.3533','E11.3539','E11.3541','E11.3542','E11.3543','E11.3549','E11.3551','E11.3552','E11.3553','E11.3559','E11.359','E11.3591','E11.3592','E11.3593','E11.3599','E11.36','E11.37X1','E11.37X2','E11.37X3','E11.37X9','E11.39','E11.40','E11.41','E11.42','E11.43','E11.44','E11.49','E11.51','E11.52','E11.59','E11.610','E11.618','E11.620','E11.621','E11.622','E11.628','E11.630','E11.638','E11.641','E11.649','E11.65','E11.69','E11.8','E13.00','E13.01','E13.10','E13.11','E13.21','E13.22','E13.29','E13.311','E13.319','E13.321','E13.3211','E13.3212','E13.3213','E13.3219','E13.329','E13.3291','E13.3292','E13.3293','E13.3299','E13.331','E13.3311','E13.3312','E13.3313','E13.3319','E13.339','E13.3391','E13.3392','E13.3393','E13.3399','E13.341','E13.3411','E13.3412','E13.3413','E13.3419','E13.349','E13.3491','E13.3492','E13.3493','E13.3499','E13.351','E13.3511','E13.3512','E13.3513','E13.3519','E13.3521','E13.3522','E13.3523','E13.3529','E13.3531','E13.3532','E13.3533','E13.3539','E13.3541','E13.3542','E13.3543','E13.3549','E13.3551','E13.3552','E13.3553','E13.3559','E13.359','E13.3591','E13.3592','E13.3593','E13.3599','E13.36','E13.37X1','E13.37X2','E13.37X3','E13.37X9','E13.39','E13.40','E13.41','E13.42','E13.43','E13.44','E13.49','E13.51','E13.52','E13.59','E13.610','E13.618','E13.620','E13.621','E13.622','E13.628','E13.630','E13.638','E13.641','E13.649','E13.65','E13.69','E13.8', |
| ***Diabetes Without Complications*** | '249','250','250.01','251.3','648','648.01','648.02','648.03','648.04','648.81','648.82','648.83','648.84', | 'E08.9','E09.9','E10.9','E11.9','E13.9','E89.1','O24.011','O24.012','O24.013','O24.019','O24.02','O24.03','O24.111','O24.112','O24.113','O24.119','O24.12','O24.13','O24.311','O24.312','O24.313','O24.319','O24.32','O24.33','O24.410','O24.414','O24.415','O24.419','O24.420','O24.424','O24.425','O24.429','O24.430','O24.434','O24.435','O24.439','O24.811','O24.812','O24.813','O24.819','O24.82','O24.83','O24.911','O24.912','O24.913','O24.919','O24.92','O24.93','O99.810','O99.814','O99.815', |
| ***Hyperlipidemia*** | '272','272.1','272.2','272.3','272.4', | 'E78.0','E78.00','E78.01','E78.1','E78.2','E78.3','E78.4','E78.41','E78.49','E78.5', |
| ***Any Other Lipid disorders*** | '272','272.1','272.2','272.3','272.4','272.5','272.6','272.7','272.8','272.9','286.53','330.1','330.2','362.71','414.3', | 'D68.312','E75.00','E75.01','E75.02','E75.09','E75.10','E75.11','E75.19','E75.21','E75.22','E75.23','E75.240','E75.241','E75.242','E75.243','E75.248','E75.249','E75.25','E75.26','E75.29','E75.3','E75.4','E75.5','E75.6','E78.0','E78.00','E78.01','E78.1','E78.2','E78.3','E78.4','E78.41','E78.49','E78.5','E78.6','E78.70','E78.71','E78.72','E78.79','E78.81','E78.89','E78.9','I25.83', |
| **Neurologic Disorders** | | |
| ***Alzheimer Disease*** | '331', | 'G30.0','G30.1','G30.8','G30.9', |
| ***Cerebrovascular disease*** | '346.6','346.61','346.62','346.63','430','431','432','432.1','432.9','433','433','433.01','433.1','433.1','433.11','433.2','433.2','433.21','433.3','433.3','433.31','433.8','433.8','433.81','433.9','433.9','433.91','434','434','434.01','434.1','434.1','434.11','434.9','434.9','434.91','435','435.1','435.2','435.3','435.8','435.9','436','437','437.1','437.2','437.3','437.4','437.5','437.6','437.7','437.8','437.9','438','438','438.1','438.11','438.12','438.13','438.14','438.19','438.2','438.21','438.22','438.3','438.31','438.32','438.4','438.41','438.42','438.5','438.51','438.52','438.53','438.6','438.7','438.81','438.82','438.83','438.84','438.85','438.89','438.9', | 'I60.00','I60.01','I60.02','I60.10','I60.11','I60.12','I60.2','I60.20','I60.21','I60.22','I60.30','I60.31','I60.32','I60.4','I60.50','I60.51','I60.52','I60.6','I60.7','I60.8','I60.9','I61.0','I61.1','I61.2','I61.3','I61.4','I61.5','I61.6','I61.8','I61.9','I62.00','I62.01','I62.02','I62.03','I62.1','I62.9','I67.1','I67.2','I67.3','I67.5','I67.6','I67.7','I67.81','I67.82','I67.83','I67.841','I67.848','I67.850','I67.858','I67.89','I67.9','I68.0','I68.2','I68.8', |
| ***Chronic Neuromuscular Disease*** | ICD-code'307.22','322','322.1','322.2','322.9','330','330.1','330.2','330.3','330.8','330.9','331','331.1','331.11','331.19','331.2','331.3','331.4','331.5','331.6','331.7','331.81','331.82','331.83','331.89','331.9','333.4','334','334.1','334.2','334.3','334.4','334.8','334.9','335.21','336.2','337.2','337.21','337.22','337.29','339.2','339.21','339.22','340','341','341.1','341.2','341.21','341.22','341.8','341.9','346.71','348','348.1','348.2','348.3','348.3','348.31','348.39','348.4','348.5','348.8','348.81','348.82','348.89','348.9','354.4','355.71','356.3','357.81','364','364.01','364.02','364.03','364.04','364.05','364.1','364.11','364.21','364.22','364.23','364.24','364.3','364.41','364.42','364.51','364.52','364.53','364.54','364.55','364.56','364.57','364.59','364.6','364.61','364.62','364.63','364.64','364.7','364.71','364.72','364.73','364.74','364.75','364.76','364.77','364.8','364.81','364.82','364.89','364.9','377.1','377.11','377.12','377.13','377.14','377.15','377.16','378.72','733.7','773.4','774.7','780.03','948.4', | ICD-code'A50.44','E75.23','E83.01','E88.42','F95.1','G03.1','G03.2','G10.','G11.4','G14.','G23.0','G23.1','G23.2','G30.0','G30.1','G30.8','G30.9','G31.01','G31.09','G31.1','G31.2','G31.81','G31.82','G31.83','G31.85','G31.89','G32.0','G32.89','G35.','G36.0','G36.8','G36.9','G37.0','G37.1','G37.2','G37.4','G37.5','G37.8','G37.9','G43.711','G43.719','G56.40','G56.41','G56.42','G56.43','G57.70','G57.71','G57.72','G57.73','G60.1','G61.81','G90.3','G90.50','G90.511','G90.512','G90.513','G90.519','G90.521','G90.522','G90.523','G90.529','G90.59','G91.4','G93.0','H47.20','H47.211','H47.212','H47.213','H47.219','H47.22','H47.231','H47.232','H47.233','H47.239','H47.291','H47.292','H47.293','H47.299','H49.40','H49.41','H49.42','H49.43','I62.03','M89.00','M89.011','M89.012','M89.019','M89.021','M89.022','M89.029','M89.031','M89.032','M89.039','M89.041','M89.042','M89.049','M89.051','M89.052','M89.059','M89.061','M89.062','M89.069','M89.071','M89.072','M89.079','M89.08','M89.09','P57.0','P57.8','P57.9','P91.1','R40.3', |
| ***Epilepsy*** | '345.1','345.1','345.4','345.4','345.5','345.5','345.9','345.9','780.39', | 'G40.309','G40.409','G40.419','G40.802','G40.811','G40.812','G40.813','G40.814','G40.821','G40.822','G40.909','G40.B01','G40.B09','R56.00','R56.01','R56.9', |
| ***Headache*** | '339.05','339.21','339.22','339.3','339.82','339.83','339.84', | 'G44.051','G44.059','G44.301','G44.309','G44.311','G44.319','G44.321','G44.329','G44.40','G44.41','G44.82','G44.83','G44.84','R51.', |
| ***Ischemic Stroke*** | '433.01','433.11','433.21','433.31','433.81','433.91','434','434.01','434.1','434.11','434.9','434.91','435.9', | 'G45.9','I63.00','I63.011','I63.012','I63.013','I63.019','I63.02','I63.031','I63.032','I63.033','I63.039','I63.09','I63.10','I63.111','I63.112','I63.113','I63.119','I63.12','I63.131','I63.132','I63.133','I63.139','I63.19','I63.20','I63.211','I63.212','I63.213','I63.219','I63.22','I63.231','I63.232','I63.233','I63.239','I63.29','I63.30','I63.311','I63.312','I63.313','I63.319','I63.321','I63.322','I63.323','I63.329','I63.331','I63.332','I63.333','I63.339','I63.341','I63.342','I63.343','I63.349','I63.39','I63.40','I63.411','I63.412','I63.413','I63.419','I63.421','I63.422','I63.423','I63.429','I63.431','I63.432','I63.433','I63.439','I63.441','I63.442','I63.443','I63.449','I63.49','I63.50','I63.511','I63.512','I63.513','I63.519','I63.521','I63.522','I63.523','I63.529','I63.531','I63.532','I63.533','I63.539','I63.541','I63.542','I63.543','I63.549','I63.59','I63.6','I63.8','I63.81','I63.89','I63.9','I66.01','I66.02','I66.03','I66.09','I66.11','I66.12','I66.13','I66.19','I66.21','I66.22','I66.23','I66.29','I66.3','I66.8','I66.9', |
| ***Myalgia*** | '74.1','710.5','729.1', | 'B33.0','M79.1','M79.10','M79.11','M79.12','M79.18', |
| ***Other Dementia*** | '290','290.1','290.11','290.12','290.13','290.2','290.21','290.3','291.2','291.2','291.21','291.22','291.23','292.82','294','294.1','294.1','294.11','294.2','294.21','294.8','331.2','797', | 'F02.80','F02.81','F03.90','F03.91','F10.27','F19.97','G10.','G31.01', |
| ***Parkinson Disease*** | '332','332.1', | 'G20.','G21.4', |
| ***Vascular Dementia*** | '290.4','290.41','290.42','290.43', | 'F01.50','F01.51', |
| **Psychiatric Disorders** | | |
| ***Alcohol Dependency*** | '291','291.1','291.2','291.3','291.4','291.5','291.8','291.81','291.82','291.89','291.9','303','303.01','303.02','303.03','303.9','303.91','303.92','303.93','305','305.01','305.02','305.03','357.5','535.3','535.3','535.31','571','571.1','571.2','571.3','760.71','980', | 'F10.10','F10.120','F10.121','F10.129','F10.14','F10.150','F10.151','F10.159','F10.180','F10.181','F10.182','F10.188','F10.19','F10.20','F10.220','F10.221','F10.229','F10.230','F10.231','F10.232','F10.239','F10.24','F10.250','F10.251','F10.259','F10.26','F10.27','F10.280','F10.281','F10.282','F10.288','F10.29','F10.920','F10.921','F10.929','F10.94','F10.950','F10.951','F10.959','F10.96','F10.97','F10.980','F10.981','F10.982','F10.988','F10.99','G31.2', |
| ***Anxiety*** | '300','300.01','300.02','300.09','300.1','300.11','300.12','300.13','300.14','300.15','300.16','300.19','300.2','300.21','300.22','300.23','300.29', | 'F40.00','F40.01','F40.02','F40.10','F40.11','F40.210','F40.218','F40.220','F40.228','F40.230','F40.231','F40.232','F40.233','F40.240','F40.241','F40.242','F40.243','F40.248','F40.290','F40.291','F40.298','F40.8','F40.9','F41.0','F41.1','F41.3','F41.8','F41.9', |
| ***Bipolar Disorder*** | '296','296.01','296.02','296.03','296.04','296.05','296.06','296.1','296.11','296.12','296.13','296.14','296.15','296.16','296.4','296.41','296.42','296.43','296.44','296.45','296.46','296.5','296.51','296.52','296.53','296.54','296.55','296.56','296.6','296.61','296.62','296.63','296.64','296.65','296.66','296.7','296.8','296.81','296.82','296.89', | 'F31.0','F31.10','F31.11','F31.12','F31.13','F31.2','F31.30','F31.31','F31.32','F31.4','F31.5','F31.60','F31.61','F31.62','F31.63','F31.64','F31.70','F31.71','F31.72','F31.73','F31.74','F31.75','F31.76','F31.77','F31.78','F31.81','F31.89','F31.9', |
| ***Drug Dependency*** | '304','304','304.001','304.002','304.003','304.009','304.01','304.01','304.011','304.012','304.013','304.019','304.02','304.02','304.021','304.022','304.023','304.029','304.03','304.03','304.031','304.032','304.033','304.039','304.09','304.1','304.1','304.101','304.102','304.103','304.104','304.105','304.106','304.107','304.108','304.109','304.11','304.11','304.111','304.112','304.113','304.114','304.115','304.116','304.117','304.118','304.119','304.12','304.12','304.121','304.122','304.123','304.124','304.125','304.126','304.127','304.128','304.129','304.13','304.13','304.131','304.132','304.133','304.134','304.135','304.136','304.137','304.138','304.139','304.14','304.15','304.16','304.17','304.18','304.19','304.2','304.21','304.22','304.23','304.3','304.3','304.309','304.31','304.31','304.319','304.32','304.32','304.329','304.33','304.33','304.339','304.39','304.4','304.4','304.401','304.409','304.41','304.41','304.411','304.419','304.42','304.42','304.421','304.429','304.43','304.43','304.431','304.439','304.49','304.5','304.5','304.509','304.51','304.51','304.519','304.52','304.52','304.529','304.53','304.53','304.539','304.59','304.6','304.6','304.609','304.61','304.61','304.619','304.62','304.62','304.629','304.63','304.63','304.639','304.7','304.71','304.72','304.73','304.8','304.81','304.82','304.83','304.9','304.9','304.909','304.91','304.91','304.919','304.92','304.92','304.929','304.93','304.93','304.939','304.99', | 'F11.20','F11.21','F11.220','F11.221','F11.222','F11.229','F11.23','F11.24','F11.250','F11.251','F11.259','F11.281','F11.282','F11.288','F11.29','F12.20','F12.21','F12.220','F12.221','F12.222','F12.229','F12.250','F12.251','F12.259','F12.280','F12.288','F12.29','F14.20','F14.21','F14.220','F14.221','F14.222','F14.229','F14.23','F14.24','F14.250','F14.251','F14.259','F14.280','F14.281','F14.282','F14.288','F14.29','F15.20','F15.21','F15.220','F15.221','F15.222','F15.229','F15.23','F15.24','F15.250','F15.251','F15.259','F15.280','F15.281','F15.282','F15.288','F15.29','F15.90','F15.920','F15.921','F15.922','F15.929','F15.93','F15.94','F15.950','F15.951','F15.959','F15.980','F15.981','F15.982','F15.988','F15.99', |
| ***Major Depressive Disorder*** | '296.2','296.21','296.22','296.23','296.24','296.25','296.26','296.3','296.31','296.32','296.33','296.34','296.35','296.36', | 'F32.0','F32.1','F32.2','F32.3','F32.4','F32.5','F32.8','F32.81','F32.89','F32.9','F33.0','F33.1','F33.2','F33.3','F33.40','F33.41','F33.42','F33.8','F33.9', |
| ***Post-traumatic stress disorder (PTSD)*** | '309.81', | 'F43.10','F43.11','F43.12', |
| ***Schizophrenia*** | '295','295.01','295.02','295.03','295.04','295.05','295.1','295.11','295.12','295.13','295.14','295.15','295.2','295.21','295.22','295.23','295.24','295.25','295.3','295.31','295.32','295.33','295.34','295.35','295.4','295.41','295.42','295.43','295.44','295.45','295.5','295.51','295.52','295.53','295.54','295.55','295.6','295.61','295.62','295.63','295.64','295.65','295.7','295.71','295.72','295.73','295.74','295.75','295.8','295.81','295.82','295.83','295.84','295.85','295.9','295.91','295.92','295.93','295.94','295.95', | 'F20.0','F20.1','F20.2','F20.3','F20.5','F20.81','F20.89','F20.9','F25.0','F25.1','F25.8','F25.9', |
| **Pulmonary Disorders** | | |
| ***Asthma*** | '493','493.01','493.02','493.1','493.11','493.12','493.2','493.21','493.22','493.81','493.82','493.9','493.91','493.92', | 'J45.20','J45.21','J45.22','J45.30','J45.31','J45.32','J45.40','J45.41','J45.42','J45.50','J45.51','J45.52','J45.901','J45.902','J45.909','J45.990','J45.991','J45.998','J82.','J82.83', |
| ***Bronchitis*** | '490','491.1','491.2','491.2','491.21','491.22','491.8','491.9', | 'J20.0','J20.1','J20.2','J20.3','J20.4','J20.5','J20.6','J20.7','J20.8','J20.9','J40.','J41.0','J41.1','J41.8','J42.','J68.0', |
| ***Chronic Lung Disease*** | '491.1','491.2','491.21','491.22','491.8','491.9','492.8','493','493.01','493.02','493.1','493.11','493.12','493.2','493.21','493.22','493.81','493.82','493.9','493.91','493.92','496','506.4','516.1','516.31','516.4','516.69','518.1','518.2','518.3','518.6','714.81','748.4','748.5','748.61','748.69', | 'B44.81','J41.0','J41.1','J41.8','J42.','J43.1','J43.2','J43.8','J43.9','J44.0','J44.1','J44.9','J45.20','J45.21','J45.22','J45.30','J45.31','J45.32','J45.40','J45.41','J45.42','J45.50','J45.51','J45.52','J45.901','J45.902','J45.909','J45.990','J45.991','J45.998','J68.4','J70.1','J81.1','J82.','J84.03','J84.112','J98.3','M05.10','M05.111','M05.112','M05.119','M05.121','M05.122','M05.129','M05.131','M05.132','M05.139','M05.141','M05.142','M05.149','M05.151','M05.152','M05.159','M05.161','M05.162','M05.169','M05.171','M05.172','M05.179','M05.19','M30.1','Q32.2','Q32.3','Q32.4','Q33.0','Q33.1','Q33.2','Q33.3','Q33.4','Q33.5','Q33.6','Q33.8','Q33.9', |
| ***Chronic Obstructive Pulmonary Disease*** | '115','115.01','115.02','115.03','115.04','115.05','115.09','115.1','115.11','115.12','115.13','115.14','115.15','115.19','115.9','115.91','115.92','115.93','115.94','115.95','115.99','490','491','491.1','491.2','491.2','491.21','491.22','491.8','491.9','492.8','494','494','494.1','496','748.61','V81.3', | 'J40.','J41.0','J41.1','J41.8','J42.','J43.0','J43.1','J43.2','J43.8','J43.9','J44.0','J44.1','J44.9','J47.0','J47.1','J47.9', |
| ***Emphysema*** | '492','492.8','518.1','518.2','770.2','958.7','998.81', | 'J43.0','J43.1','J43.2','J43.8','J43.9','J98.2','J98.3','P25.0','P25.8','T79.7XXA','T79.7XXD','T79.7XXS','T81.82XA','T81.82XD','T81.82XS', |
| ***Pulmonary Hypertension Disorder*** | '114.4','114.5','397.1','415.1','415.12','415.13','415.19','416.2','416.8','416.9','417.1','417.8','417.9','424.3','506.1','508.1','514','515','516.1','516.2','516.31','516.5','516.62','518.3','518.5','518.52','518.82','786.31','793.11','794.2','V12.55', | 'I27.0','I27.1','I27.2','I27.20','I27.21','I27.22','I27.23','I27.24','I27.29','I27.81','I27.82','I27.83','I27.89','I27.9','I28.0','I28.1','I28.8','I28.9', |
| ***Pulmonary Embolism*** | '415.1','415.11','415.12','415.19','416.2','673.8','673.81','673.82','673.83','673.84','V12.55', | 'I26.02','I26.09','I26.92','I26.93','I26.94','I26.99', |
| ***Pulmonary Fibrosis*** | '515','516.31', | 'J63.1','J84.10','J84.112','J84.17','J84.178','J84.89', |
| ***Venous Thromboembolism*** | '415.11','415.12','415.13','415.19','451.11','451.19','451.81','451.83','453.2','453.4','453.41','453.42','453.82','453.83','453.84','453.85','453.86','453.87', | 'I26.01','I26.02','I26.09','I26.90','I26.92','I26.93','I26.94','I26.99','I80.10','I80.11','I80.12','I80.13','I80.201','I80.202','I80.203','I80.209','I80.211','I80.212','I80.213','I80.219','I80.221','I80.222','I80.223','I80.229','I80.231','I80.232','I80.233','I80.239','I80.241','I80.242','I80.243','I80.249','I80.251','I80.252','I80.253','I80.259','I80.291','I80.292','I80.293','I80.299','I82.210','I82.220','I82.290','I82.401','I82.402','I82.403','I82.409','I82.411','I82.412','I82.413','I82.419','I82.421','I82.422','I82.423','I82.429','I82.431','I82.432','I82.433','I82.439','I82.441','I82.442','I82.443','I82.449','I82.451','I82.452','I82.453','I82.459','I82.461','I82.462','I82.463','I82.469','I82.491','I82.492','I82.493','I82.499','I82.4Y1','I82.4Y2','I82.4Y3','I82.4Y9','I82.4Z1','I82.4Z2','I82.4Z3','I82.4Z9','I82.621','I82.622','I82.623','I82.629','I82.A11','I82.A12','I82.A13','I82.A19','I82.B11','I82.B12','I82.B13','I82.B19','I82.C11','I82.C12','I82.C13','I82.C19', |
| **Renal Disorders** | | |
| ***Acute Kidney Failure*** | '584.5','584.6','584.7','584.8','584.9','639.3','669.32','669.34', | 'N17.0','N17.1','N17.2','N17.8','N17.9','N19.','N99.0','O90.4', |
| ***Chronic Kidney disease*** | '285.21','361.04','364.76','403.01','403.1','403.11','403.9','403.91','404.01','404.02','404.03','404.1','404.11','404.12','404.13','404.9','404.91','404.92','404.93','458.21','585','585.1','585.2','585.3','585.4','585.5','585.6','585.9','753.1','753.19','792.5','996.56','996.68','996.73','V18.6','V18.69','V45.1','V45.11','V45.12','V56.0','V56.1','V56.2','V56.31','V56.32','V56.8', | 'D63.1','E08.22','E09.22','E10.22','E11.22','E13.22','I12.0','I12.9','I13.0','I13.10','I13.11','I13.2','N18.1','N18.2','N18.3','N18.30','N18.31','N18.32','N18.4','N18.5','N18.6','N18.9','R88.0','Z49.01','Z49.02','Z49.31','Z49.32', |
| ***Chronic Kidney Failure*** | '403.01','403.11','403.91','404.02','404.03','404.12','404.13','404.92','404.93','585.5','585.6', | 'I12.0','I13.11','I13.2','N18.6','N99.0', |
| ***Dialysis*** | '585.6','792.5','V45.1','V45.11','V45.12','V56.0','V56.1','V56.2','V56.31','V56.32','V56.8', | 'N18.6','R88.0','Z49.01','Z49.02','Z49.31','Z49.32','Z91.15','Z99.2', |
| ***Other Kidney conditions*** | '16','16.01','16.02','16.03','16.04','16.05','16.06','16.1','16.11','16.12','16.13','16.14','16.15','16.16','16.2','16.21','16.22','16.23','16.24','16.25','16.26','16.3','16.31','16.32','16.33','16.34','16.35','16.36','16.4','16.41','16.42','16.43','16.44','16.45','16.46','16.5','16.51','16.52','16.53','16.54','16.55','16.56','16.6','16.61','16.62','16.63','16.64','16.65','16.66','16.7','16.71','16.72','16.73','16.74','16.75','16.76','16.9','16.91','16.92','16.93','16.94','16.95','16.96','160.1','160.2','160.3','160.4','160.5','189','189.1','189.2','189.3','189.4','189.8','189.9','198','198.1','198.2','198.3','198.4','198.5','198.6','198.7','198.81','198.82','198.89','209.24','209.64','223','223.1','223.2','223.3','223.81','223.89','223.9','236.91','274.1','274.11','274.19','285.21','403.01','403.1','403.11','403.9','403.91','404.01','404.02','404.03','404.1','404.11','404.12','404.13','404.9','404.91','404.92','404.93','445.81','580.4','580.81','580.89','580.9','581.1','581.2','581.3','581.81','581.89','581.9','582.1','582.2','582.4','582.81','582.89','582.9','583.1','583.2','583.4','583.6','583.7','583.81','583.89','583.9','584.5','584.6','584.7','584.8','584.9','585.1','585.2','585.3','585.4','585.5','585.9','588.1','589.1','589.9','590.01','590.11','590.2','590.81','590.9','591','593.1','593.2','593.7','593.71','593.72','593.73','593.81','593.89','593.9','639.3','669.32','669.34','753.1','753.12','753.13','753.14','753.16','753.17','753.19','753.3','794.4','866.01','866.02','866.03','866.11','866.12','866.13','954','954.1','954.8','954.9','969.02','996.81','V10.52','V13.03','V16.51','V18.6','V18.61','V18.69','V42.0','V45.73','V59.4','V81.5', | 'A18.11','A36.84','A51.44','A54.21','B26.83','B52.0','B58.83','D63.1','E08.21','E08.22','E09.21','E09.22','E10.21','E10.22','E11.21','E11.22','E13.21','E13.22','I12.0','I12.9','I13.0','I13.10','I13.11','I13.2','N00.0','N00.1','N00.2','N00.3','N00.4','N00.5','N00.6','N00.7','N00.8','N00.9','N01.0','N01.1','N01.2','N01.3','N01.4','N01.5','N01.6','N01.7','N01.8','N01.9','N02.0','N02.1','N02.2','N02.3','N02.4','N02.5','N02.6','N02.7','N02.8','N02.9','N03.0','N03.1','N03.2','N03.3','N03.4','N03.5','N03.6','N03.7','N03.8','N03.9','N04.0','N04.1','N04.2','N04.3','N04.4','N04.5','N04.6','N04.7','N04.8','N04.9','N05.0','N05.1','N05.2','N05.3','N05.4','N05.5','N05.6','N05.7','N05.8','N05.9','N06.0','N06.1','N06.2','N06.3','N06.4','N06.5','N06.6','N06.7','N06.8','N06.9','N07.0','N07.1','N07.2','N07.3','N07.4','N07.5','N07.6','N07.7','N07.8','N07.9','N08.','N13.0','N13.1','N13.2','N13.30','N13.39','N13.4','N13.5','N13.8','N13.9','N14.0','N14.1','N14.2','N14.3','N14.4','N15.0','N15.8','N15.9','N16.','N17.0','N17.1','N17.2','N17.8','N17.9','N18.1','N18.2','N18.3','N18.4','N18.5','N18.6','N18.9','N19.','N20.0','N20.1','N20.2','N20.9','N21.0','N21.1','N21.8','N21.9','N22.','N23.','N25.0','N25.1','N25.81','N25.89','N25.9','N26.1','N26.2','N26.9','N27.0','N27.1','N27.9','N28.0','N28.1','N28.81','N28.82','N28.83','N28.84','N28.85','N28.86','N28.89','N28.9','N29.','N99.0','O26.831','O26.832','O26.833','O26.839','O90.4','R88.0','Z49.01','Z49.02','Z49.31','Z49.32', |
| ***Nephrosis*** | '274.1','274.11','274.19','403.01','403.1','403.11','403.9','403.91','404.02','404.03','404.1','404.12','404.13','404.9','404.92','404.93','580.4','580.81','580.89','580.9','581.1','581.2','581.3','581.81','581.89','581.9','582.1','582.2','582.4','582.81','582.89','582.9','583.1','583.2','583.4','583.6','583.7','583.81','583.89','583.9','585.6','590.01','590.11','590.2','590.81','591','593.6','593.7','593.71','593.72','593.73','599.7','599.71','599.72','642.11','642.12','642.13','642.14','646.21','646.22','646.23','646.24','791','791.1','791.2','791.3','791.4','791.5','791.6','791.7','791.9','V13.03','V81.5', | 'A36.84','A51.44','B26.83','B52.0','B58.83','E08.21','E09.21','E10.21','E11.21','E13.21','N00.0','N00.1','N00.2','N00.3','N00.4','N00.5','N00.6','N00.7','N00.8','N00.9','N01.0','N01.1','N01.2','N01.3','N01.4','N01.5','N01.6','N01.7','N01.8','N01.9','N02.0','N02.1','N02.2','N02.3','N02.4','N02.5','N02.6','N02.7','N02.8','N02.9','N03.0','N03.1','N03.2','N03.3','N03.4','N03.5','N03.6','N03.7','N03.8','N03.9','N04.0','N04.1','N04.2','N04.3','N04.4','N04.5','N04.6','N04.7','N04.8','N04.9','N05.0','N05.1','N05.2','N05.3','N05.4','N05.5','N05.6','N05.7','N05.8','N05.9','N06.0','N06.1','N06.2','N06.3','N06.4','N06.5','N06.6','N06.7','N06.8','N06.9','N07.0','N07.1','N07.2','N07.3','N07.4','N07.5','N07.6','N07.7','N07.8','N07.9','N08.','N14.0','N14.1','N14.2','N14.3','N14.4','N15.0','N15.8','N15.9','N16.','N26.9', |
| ***Urinary Stone*** | '274.11','574.01','574.11','574.21','574.31','574.41','574.51','574.61','574.71','574.81','574.91','592.1','592.9','594','594.1','594.2','594.8','594.9','602','V13.01', | 'A18.11','A54.21','N13.0','N13.1','N13.2','N13.30','N13.39','N13.4','N13.5','N13.8','N13.9','N20.0','N20.1','N20.2','N20.9','N21.0','N21.1','N21.8','N21.9','N22.','N23.','N25.0','N25.1','N25.81','N25.89','N25.9','N26.1','N26.2','N27.0','N27.1','N27.9','N28.0','N28.1','N28.81','N28.82','N28.83','N28.84','N28.85','N28.86','N28.89','N28.9','N29.','O26.831','O26.832','O26.833','O26.839', |

| **Table S5.** Adjusted Odds Ratios (95% confidence intervals) for all-cause mortality associated with sleep apnea among the entire cohort and subgroups of PSG and HST. | | | |
| --- | --- | --- | --- |
| **Adj. OR (95%CI)** | | | |
|  | **Entire Cohort N=146,148** | **PSG**  **N= 64,652** | **HST**  **N=81,496** |
| **All ages** | | | |
| n-SA (AHI < 5) | REF |  |  |
| s-SA (AHI ≥ 30) | 0.56 (0.54, 0.58) | 0.79(0.75,0.83) | 0.76(0.7,0.81) |
| **Age ≤ 40** | | | |
| n-SA (AHI < 5) | REF |  |  |
| s-SA (AHI ≥ 30) | 1.11 (0.93, 1.32) | 1.5(1.18,1.91) | 1.17(0.89,1.53) |
| **40<age<65** | | | |
| n-SA (AHI < 5) | REF |  |  |
| s-SA (AHI ≥ 30) | 0.64 (0.61, 0.67) | 0.91(0.86,0.97) | 0.81(073,0.9) |
| **Age ≥ 65** | | | |
| n-SA (AHI < 5) | REF |  |  |
| s-SA (AHI ≥ 30) | 0.44 (0.41, 0.46) | 0.59(0.54,0.63) | 0.63(0.57,0.71) |
| Adjusted for age, sex, race, ethnicity, BMI and CCI. | | | |
